# Uncovering the diversity and distribution of biosynthetic gene clusters of prochlorosins and other putative RiPPs in marine *Synechococcus* strains

**DOI:** 10.1128/spectrum.03611-23

**Published:** 2023-12-13

**Authors:** Patricia Arias-Orozco, Lu Zhou, Yunhai Yi, Rubén Cebrián, Oscar P. Kuipers

**Affiliations:** 1 Department of Molecular Genetics, University of Groningen, Nijenborgh, Groningen, The Netherlands; 2 Department of Plants and Crops, Faculty of Bioscience Engineering, Ghent University, Ghent, Belgium; 3 Department of Clinical Microbiology, Instituto de Investigación Biosanitaria ibs.GRANADA, San Cecilio University Hospital, Granada, Spain; 4 CIBER de Enfermedades Infecciosas, CIBERINFEC, ISCIII, Madrid, Spain; University of Melbourne, Melbourne, Australia

**Keywords:** *Synechococcus*, lanthipeptides, prochlorosins, synechococsins

## Abstract

Picocyanobacteria can synthesize a distinctive class of lanthipeptides referred to as prochlorosins. They are classified within the Nif11 family and are subject to post-translational modifications by a solitary promiscuous enzyme from the ProcM group. Their biological function is unknown but highly speculated upon. In this study, we conducted a comprehensive genome mining investigation of the *Synechococcus* and *Prochlorococcus* genomes to elucidate the genomic landscape associated with the biosynthetic gene clusters (BGCs) harboring prochlorosin genes. Our mining study led to a substantial expansion of the known prochlorosin repertoire identified in 15 picocyanobacterial genomes. We also identified a range of tailoring enzymes and gene families proximal to the *procA*/*syncA* genes. The recombination-related proteins we detected were of particular interest, as they may have a critical role in generating the diversity observed within this peptide family. Interestingly, we identified novel LAP/YcaO BGCs in *Synechococcus* that had not been previously described. These BGCs hold the potential to generate prochlorosins with secondary modifications and introduce new putative azol(in)e-containing ribosomally synthesized and post-translationally modified peptides precursors. Our investigation offers an in-depth analysis of the vast prochlorosin family.

**IMPORTANCE:**

Genome mining studies have revealed the remarkable combinatorial diversity of ribosomally synthesized and post-translationally modified peptides (RiPPs) in marine bacteria, including prochlorosins. However, mining strategies also prove valuable in investigating the genomic landscape of associated genes within biosynthetic gene cluster (BGC) specific to targeted RiPPs of interest. Our study contributes to the enrichment of knowledge regarding prochlorosin diversity. It offers insights into potential mechanisms involved in their biosynthesis and modification, such as hyper-modification, which may give rise to active lantibiotics. Additionally, our study uncovers putative novel promiscuous post-translational enzymes, thereby expanding the chemical space explored within the *Synechococcus* genus. Moreover, this research extends the applications of mining techniques beyond the discovery of new RiPP-like clusters, allowing for a deeper understanding of genomics and diversity. Furthermore, it holds the potential to reveal previously unknown functions within the intriguing RiPP families, particularly in the case of prochlorosins.

## INTRODUCTION

The marine picocyanobacteria *Synechococcus* and *Prochlorococcus* are the most abundant phototrophs in the global oceans (1 – 3). *Synechococcus* is widely distributed, inhabiting diverse freshwater and marine environments, including polar regions and high-nutrient waters (1, 4). *Prochlorococcus* extends to deep marine water (~200 m) and is more niche-restricted than *Synechococcus*. In high-nutrient waters, *Prochlorococcus* can be outcompeted by other phytoplankton species (1, 4). Various studies have described the diversity of their natural products with vast biotechnological potential, including therapeutic applications (5 – 9). These secondary metabolites play critical roles in the marine ecosystem’s biogeochemical cycles (i.e., carbon cycles) and cellular processes such as metal transport, cell-to-cell signaling, and defense systems (10). Moreover, the synthesis of such a diverse range of molecules could provide distinct benefits by offering a selective advantage against competing organisms or aiding in adaptation to environmental changes (11). The biosynthetic pathways of these secondary metabolites can be ribosomal (gene encoded), non-ribosomal peptide synthetase-based, or polyketide synthase-based (PKS) (5, 10). These last two synthesis routes need a large complex of multi-modular enzymatic assembly lines (5).

**Fig 1:**
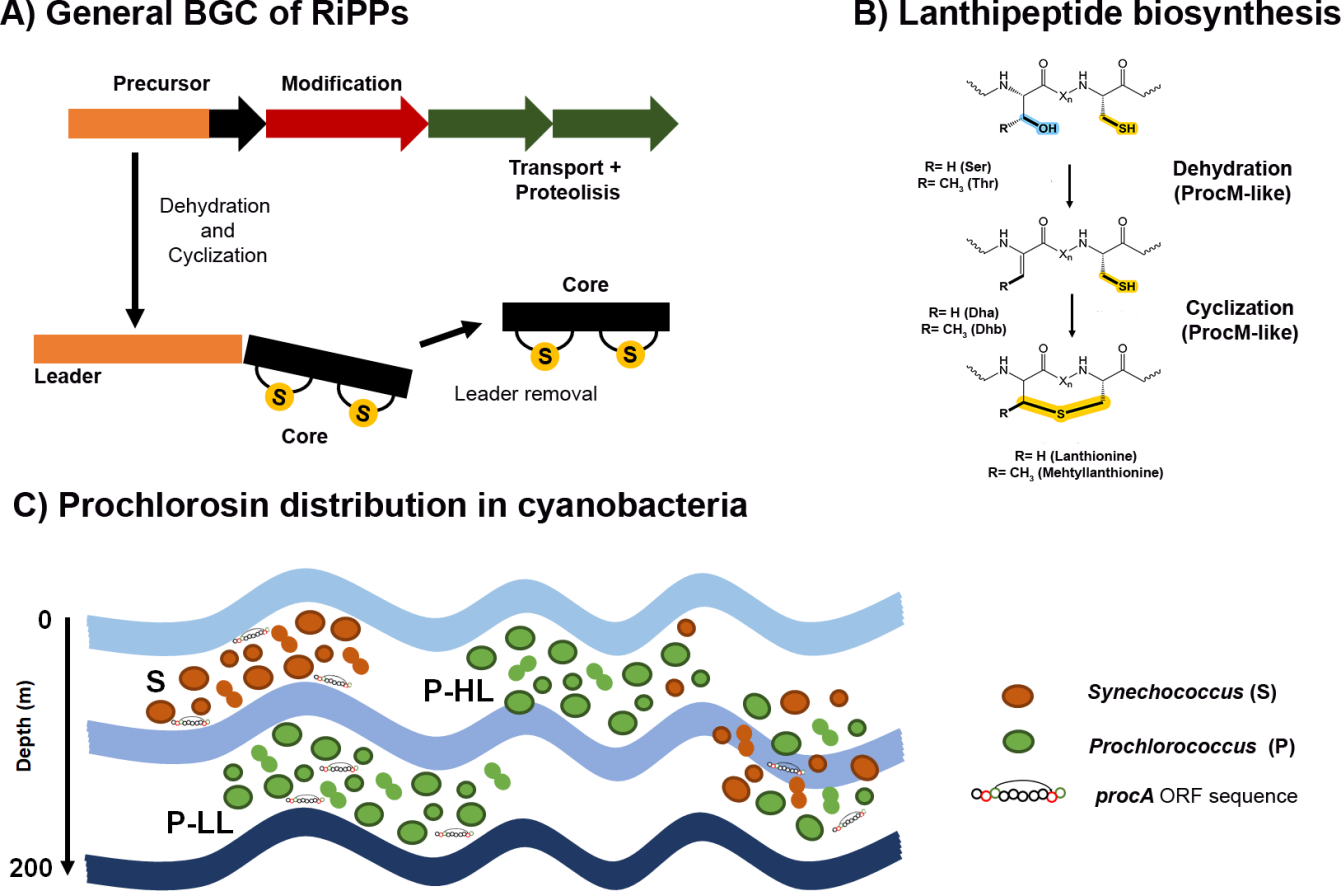
General description of BGCs and class II lanthipeptide biosynthesis. (**A**) An illustration of a biosynthetic gene cluster belonging to the class II lanthipeptide category. (**B**) Dehydration and cyclization reactions involved in the biosynthesis of class II lanthipeptides. The modification of prochlorosins is facilitated by ProcM-like enzymes. (**C**) The distribution of the ProcA/SyncA open reading frame (ORF) sequences in various oceanic locations within the wild populations of *Synechococcus* and *Prochlorococcus* (3). The presence of two *Prochlorococcus* ecotypes, namely, P-LL (low-light adapted) and P-HL (high-light adapted), is indicated. Bacteria containing the *procA* and *syncA* genes are found at low/high altitudes in the Atlantic Ocean, where *Synechococcus* dominates. *Prochlorococcus* low-light adapted ecotype thrives in deeper waters. In deep water, a homogeneous mixing event between P-LL and S (*Synechococcus*) can occur (3).

The ribosomally synthesized and post-translationally modified peptides (RiPPs) form a large group of secondary metabolites (12). Their general BGC (Fig. 1A) is composed of a gene that encodes a precursor peptide spliced in two parts; a core peptide, in which post-translational modifications (PTMs) occur, and a leader peptide with a recognition motif for one or more post-translational modification enzymes (13, 14). Other roles have been described, such as keeping the precursor peptide inactive until secretion, assisting in precursor folding and stabilization, and /or ensuring that PTMs occur in the correct order (13, 15). Additionally, BGCs encompass genes encoding enzymes for PTMs, proteins involved in transport and proteolysis and, in some cases, immunity genes (Fig. 1A).

Marine-RiPPs represent a unique class of natural products with distinctive combinatorial biosynthesis. These peptides often exhibit hypervariable sequences and are encoded within multi-modular BGCs that house enzymes with extremely relaxed substrate specificity (2). Due to the diverse topologies and modifications present in a single precursor, marine-RiPPs have garnered significant attention as promising targets for peptide engineering and synthetic biology approaches. These strategies aim to discover or produce novel molecules with diverse biotechnological applications (14, 16). The advent of genome sequencing and advanced bioinformatic tools has revolutionized RiPP mining studies, which are now widely conducted to identify novel BGCs associated with various natural products, including antimicrobial agents (17 – 20). Mining efforts have unveiled the existence of multiple lanthipeptide-type RiPPs in cyanobacteria, particularly in picocyanobacteria (7, 8, 21).

Lanthipeptides constitute a significant subfamily of RiPPs distinguished by dehydrated serine and/or threonine residues and one or more (β-methyl) lanthionine rings (Fig. 1B). In Class II lanthipeptides, the bifunctional enzyme LanM catalyzes the initial dehydration of serine/threonine residues, followed by cyclization between dehydroalanine (Dha)/dehydrobutyrine (Dhb) and a cysteine residue (13, 22) (Fig. 1A and B). The prochlorosin family is an example of this class, characterized by hypervariable precursors and belonging to a subclass of cyanobacterial peptides known as Nif11, derived from the nitrogen fixation family (8). While lanthipeptide synthetases often exhibit selectivity toward a specific precursor peptide (or occasionally two), this is not the case for the prochlorosin family (2, 10). Instead, a single promiscuous enzyme from the ProcM-like group possesses a broad-range substrate tolerance and catalytic mechanism, enabling the modification of multiple ProcA/SyncA precursors (Fig. 1B) (3, 10). Currently, two ProcM-like enzymes have been characterized. The first is ProcM from *Prochlorococcus* MIT9313, responsible for modifying 29 distinct substrates (ProcA) (10). The second is SyncM from *Synechococcus* MIT9509, associated with 79 putative precursors known as synechococsins (SyncA) (23). The biosynthesis genes for prochlorosins (*procA*/*syncA*) exhibit genomic flexibility, as they can be scattered across the genome. They may be found within complete BGCs containing *lan*M, *lan*TP, and *lan*OM genes or as individual precursor genes surrounded by different gene families (3, 23).

Prochlorosins are widespread across different marine environments (Fig. 1C) (3, 10). However, their phylogenetic distribution appears to be limited, as they have been identified only in the low-light-adapted branch clade LLIV of *Prochlorococcus* (*procA*), which is abundant in deep waters (Fig. 1C) (3, 10). In the case of *Synechococcus* (*syncA*), they are found in distantly related clades (I, IX, UC-A, and CRD1) (3) within the 5.1 submarine cluster (3), which predominates in open ocean waters (24). Through metagenomic analysis of oceanic water samples, numerous open reading frame (ORF) sequences of *procA*/*syncA* were identified, exhibiting variations in core peptide amino acid sequences (Fig. 1C) (3). Similarly, a recent metagenomic study in freshwater samples detected prochlorosin ORF sequences (25). Furthermore, in laboratory conditions, the expression of *procA* reaches its maximum level during the exponential phase of *Prochlorococcus* MIT9313 (10). These observations suggest that prochlorosins play an integral role in cyanobacterial populations. However, their specific biological function remains unknown.

To explore and unravel the diversity of BGCs harboring *procA* or *syncA* genes, we conducted mining of 38 *Synechococcus* and 9 *Prochlorococcus* cyanobacterial genomes. Among the cyanobacterial genomes analyzed, the *Synechococcus* genus within clade CDRI exhibited the highest abundance of putative synechococsins. We also discovered additional enzymes involved in post-translational modifications in close proximity to the *procA*/*syncA* genes. Furthermore, we observed diverse genomic contexts encompassing proteins associated with cellular processes, transport, and regulation. Notably, we present the identification and description of a novel group of BGCs containing the *yca*O gene, which has not been previously reported in the *Synechococcus* genus. These gene clusters can potentially introduce novel secondary modifications in prochlorosins and yield unique azol(in)e-containing RiPP peptides. Surprisingly, our study identified up to 484 putative ProcAs/SyncAs, significantly expanding the prochlorosin library beyond the previously described 181 sequences (3). These findings provide valuable insights into the diversity and biosynthesis of prochlorosins and related peptides in cyanobacteria, thereby facilitating the discovery of new RiPPs.

## RESULTS

### Prediction of RiPPs-encoding BGCs in marine *Synechococcus* and *Prochlorococcus*

Our study involved the analysis of genomes from 38 marine *Synechococcus* and 9 *Prochlorococcus* strains, with the data collected in 2019. The selected genomes were chosen from distantly related clades (3, 4) (Table S1) and specifically included strains known to contain prochlorosins (3, 23). The BGCs responsible for the production of ribosomally synthesized peptides were identified using the AntiSMASH 5.0 platform. This bioinformatic tool utilizes a rule-based detection approach to identify 52 types of BGCs, identifying conserved core enzymes and classifying them into BGCs (26). In total, we analyzed 287 putative RiPP biosynthetic clusters from these genomes, of which 81 contained *procA*/*syncA*-like genes and 206 BGCs encoded other Nif11-related precursors (Table S1) (8, 21).

**Fig 2:**
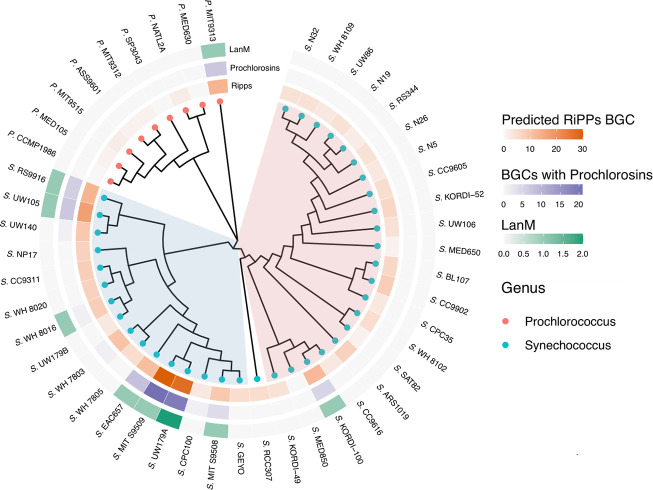
Phylogenetic tree of the marine *Synechococcus* and *Prochlorococcus* genomes used for mining analysis. The inner to outer rings represent the number of predicted BGCs (orange), BGCs containing putative prochlorosin precursors (purple), and LanM enzymes (putative ProcM-like enzymes) (green). Clade I (not shadowed) consists of *Prochlorococcus* genomes, with only *P. MIT9313* strain encoding *procA*-BGC. Clade II (shadowed in blue): this clade comprises *Synechococcus* genomes with the highest number of BGCs containing putative synechococsins. Clade III (shadowed in red): this clade also consists of *Synechococcus* genomes; however, only *S*. KORDI-100 has s*yncA*-BGCs. See legend on the right.

Based on the phylogenetic tree in Fig. 2, our analysis revealed three distinct main clades. One clade consisted of *Prochlorococcus*, with the only prochlorosin-producing strain, *Prochlorococcus* MIT9313 (LLIV), adapted to low-light conditions. This group exhibited the lowest number of identified RiPP BGCs (Fig. 2). In contrast, the *Synechococcus* genomes were divided into two separate clades within the phylogenetic tree. Notably, the second clade exhibited a higher prevalence of BGCs encoding putative SyncA precursors and LanM enzymes (Fig. 2). Among the *Synechococcus* strains, *S*. MIT9509 (CDRI) and *S*. UW179A (CDRI) displayed the highest number of putative prochlorosin precursors, with 83 and 79 sequences, respectively. *S*. EAC 657 was also found in close proximity to them, followed by distantly related strains *S*. RS9116 (IX) and *S*. UW105 (XVI). In the third clade, only *S*. KORDI-100 exhibited *syncA* genes.

Furthermore, consistent with previous studies (8, 21), the Nif11 family peptides were the most prevalent class of ribosomally synthesized precursors in the *Synechococcus* and *Prochlorococcus* genomes. However, prochlorosins were identified in only a limited number of genomes (12). Notably, the *Synechococcus* genomes of the CDRI clade (*S*. MIT9509, *S*. UW179A) exhibited the highest number of *syncA* genes, suggesting their significance in this specific clade.

### BGC similarity network: identification of putative lanthipeptides (Prochlorosin) and other Nif11 peptide precursor family clusters

To investigate the genomic distribution and diversity of RiPP BGCs associated with the prochlorosin family and other Nif11-related precursors, we employed the BiG-SCAPE platform. This tool utilizes sequence similarity to create a network of BGCs and classify them into gene cluster families (GCFs) (27).

**Fig 3:**
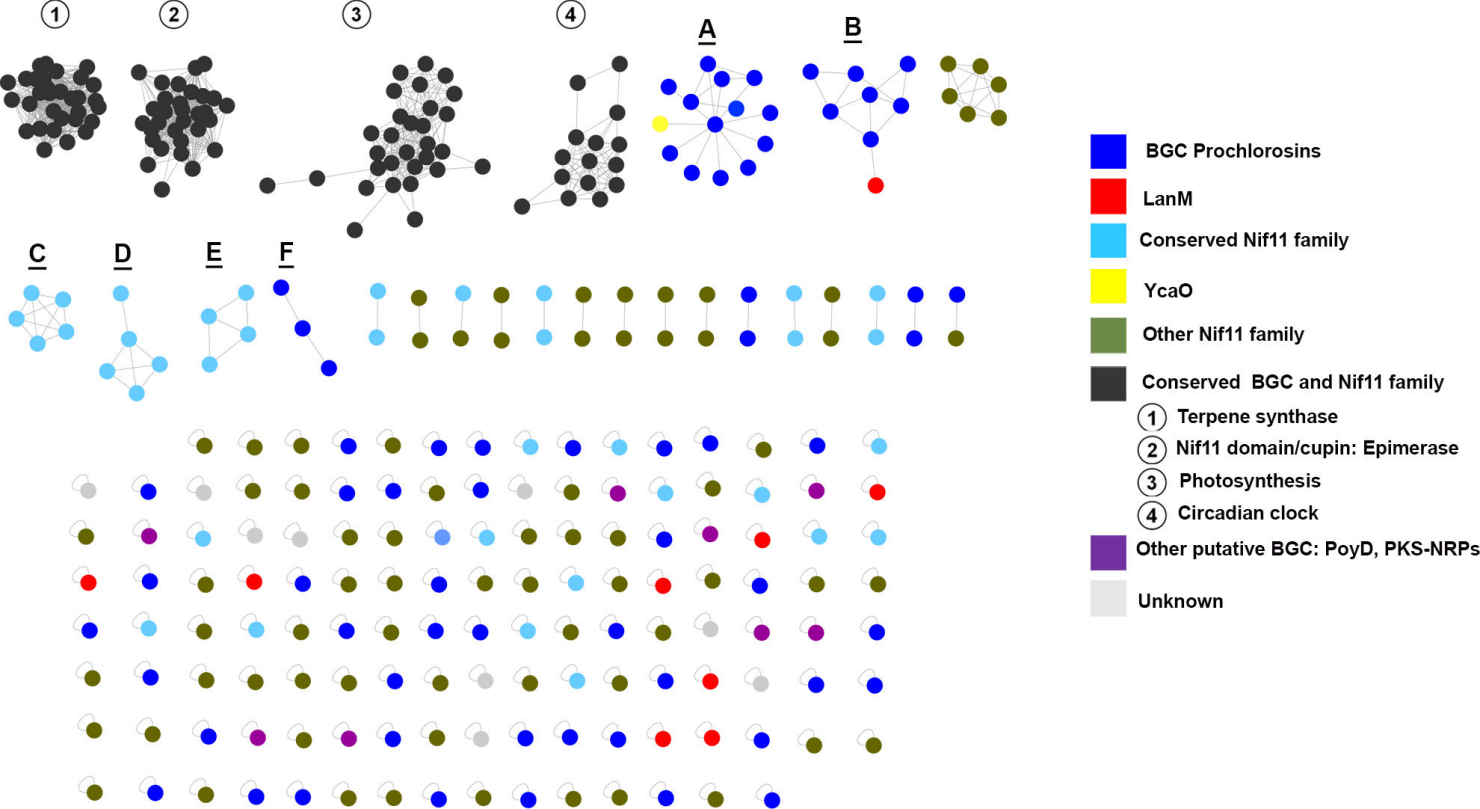
BGC similarity network generated by BIG-SCAPE. Each node within the network represents a unique BGC clustered within the GCFs. The color of each BGC node corresponds to the identified precursors present in the Nif11 family’s BGCs, other putative BGSs, or unknown. For example, blue nodes denote clusters with *procA/syncA* genes, while red nodes signify the presence of LanM enzymes (ProcM-like). Purple nodes represent BGC associated with other natural product families. The legend on the right provides a key for each color.

The generated network, presented in Fig. 3, depicts the clustering of various Nif11-like leader peptide precursors into distinct clusters. Among these clusters, 81 BGCs contained putative *procA* and *syncA* genes. Additionally, over half (59.8%) of the BGCs exhibited interactions with other BGCs, while 36.6% of the BGCs appeared as singletons (individual nodes). Clusters 1–4 were characterized by different Nif11 precursors (represented by black nodes) within conserved BGCs (Fig. S1). Cluster 1 featured a conserved terpene synthetase, while cluster 2 included a Nif11 domain/cupin with a putative epimerase tailoring enzyme, suggesting the presence of D-amino acids in the peptide. Clusters 3 and 4 encompass Nif11 family substrate precursors located near genes associated with the photosynthetic family and circadian clock-related genes, respectively. Furthermore, a conserved distribution of the Nif11 family was observed across both marine families (illustrated by light blue nodes, Fig. 3) and grouped into clusters C, D, and E (Fig. S2) in 5 doubles and 14 singletons. This analysis identified various Nif11 precursors (Fig. S3), including prochlorosins/synechococsins precursors with a slight conserved N-terminal (-MS/TEEQL-), the characteristic C-terminal motif (-D/EELExxxGG-), and a non-conserved core peptide. Furthermore, examples in Fig. S3 show other Nif11 leader precursors with different N-terminal and C-terminal sequences. For instance, in group F, we observed a N-terminal sequence (-MALDQL-) and a putative C-terminal motif (-D/ELL) with mostly a conserved core peptide at the C-terminal (-GEYH/N-). The function of this family of peptides is still unknown.

In the analysis, three gene cluster families were identified (Fig. 3: groups A, B, and F) that encompass BGCs encoding *procA*/*syncA* genes (blue). These GCFs exhibit multiple interactions, with more than three BGCs interacting within each group and five BGCs interacting in pairs. Other prochlorosin BGCs are distributed as single nodes (28). Among the prochlorosin-containing strains, LanM enzymes (red, Fig. 3) are present in 9 of the 12 strains. Group B represents the only case where a BGC with a ProcM-like enzyme is observed. The remaining ProcM-like enzymes are distributed as singletons without interacting with other BGCs in the genomes.

#### Identification of ProcM-like enzymes and putative prochlorosins precursors

**Fig 4:**
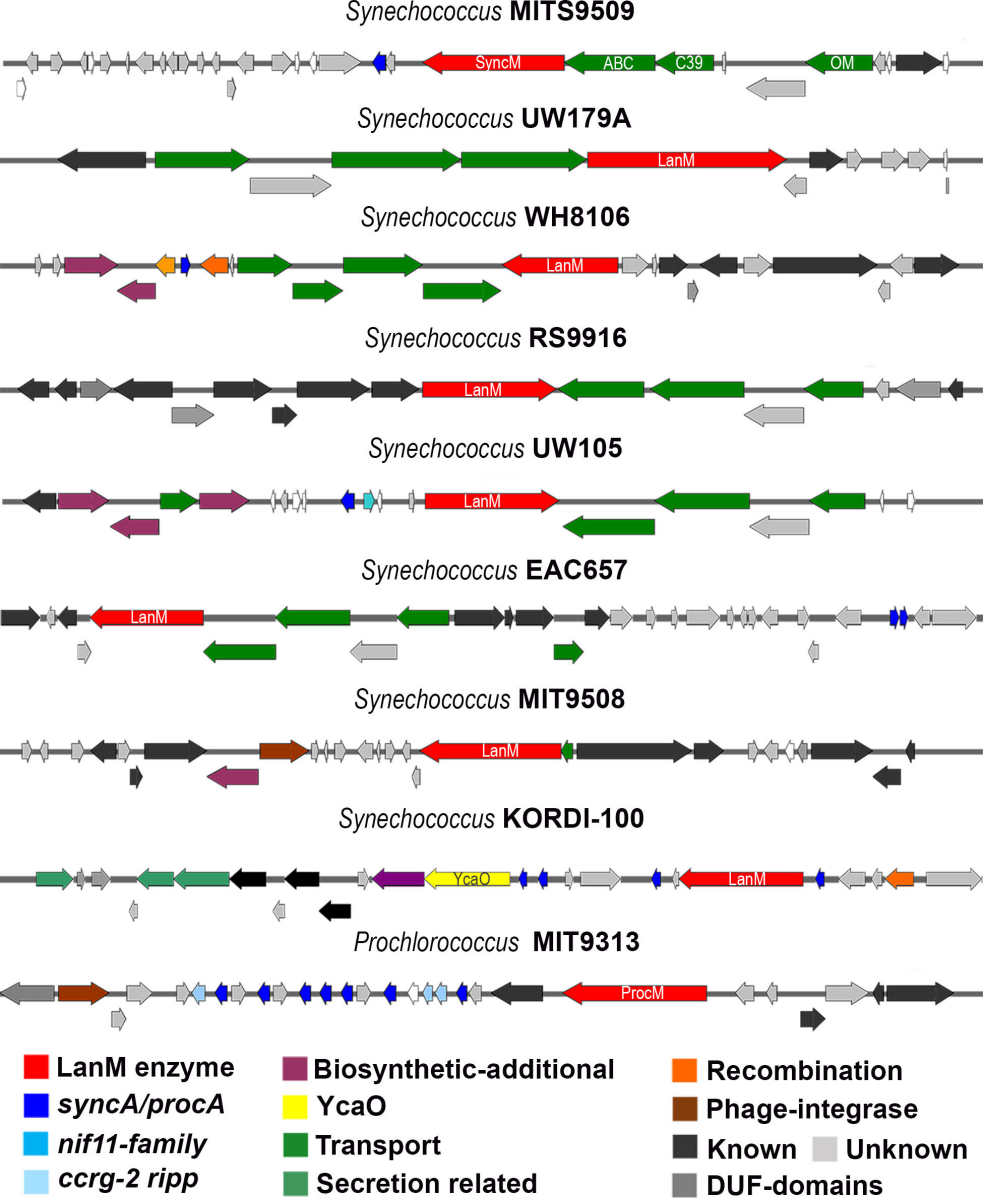
BGCs with putative ProcM-like enzymes identified with AntiSMASH. Nine ProcM-like enzymes were specifically detected within these BGCs. Additionally, putative tailoring enzymes were observed in proximity to these genes. Among them, the YcaO enzyme was identified, which is known to be involved in post-translational modifications related to the heterocyclization of cysteine (Cys), threonine (Thr), and serine (Ser) residues. It is worth noting that these additional PTM enzymes may or may not be directly associated with the prochlorosin biosynthetic machinery.

In Fig. 4, we describe the different BGCs containing ProcM-like enzymes. Through alignment analysis, it was observed that all identified LanM enzymes in these BGCs possess the characteristic “CCG” motif associated with the ProcM-like lanthipeptide synthetase family (29) (see Fig. S4). This motif consists of three zinc-binding sites proposed to contribute to the enzyme’s promiscuity (22). Among the 12 strains with at least one putative *procA*/*syncA* gene, 9 strains were found to encode a LanM (represented by red nodes) lanthipeptide synthetase (Table S1). In the vicinity of lanthipeptide-related genes in these *Synechococcus* strains (Fig. 4), various transport-related proteins (green) were observed, including the TolC family, HylD, ABC-transporters, C39 protease, and outer membrane efflux protein. In the case of *S*. KORDI-100, a PTM enzyme called YcaO, associated with the TOMM family (thiazole/oxazole-modified microcin), was identified. This cluster also encompasses a SagB-like dehydrogenase. These two enzymes are part of the standard azole and azoline heterocycle synthesis machinery, described in azole-containing peptides in cyanobactins. They will be discussed in detail in the following section (30, 31). Interestingly, another potential PTM related to the RiPP modification machinery was identified, such as a SAM-dependent methyltransferase (32) in *S*. UW105 and a NAD(P)/FAD-dependent oxidoreductase in *S*. WH8106. These enzymes may play a role in the modification of RiPPs and contribute to the diversity of post-translational modifications observed in these strains.

In *Synechococcus* UW179A and *Prochlorococcus* MIT9313, a higher number of *syncA*/*procA* precursor genes were identified than initially expected (Table S1). This observation can be attributed to the continuous improvement and updating of bioinformatic tools used for genome mining, which enhance the detection of RiPPs based on updated annotations. To validate the findings, we compared the detection results of AntiSMASH 5.0 with BAGEL4 (33) and BLASTp. Combining the results from all platforms and manual curation, we assembled a comprehensive list of identified prochlorosins (Table S1).

Sequence logo analysis (Fig. S5) of the putative prochlorosins from each strain revealed a high degree of conservation in the N-terminal leader peptide and the C-terminal double Gly-Gly motif, which is involved in transport and cleavage processes. However, the C-terminal core peptide exhibited lower conservation, even among a limited number of precursor peptides (Fig. S5). Notably, certain cysteine positions within the core peptide showed relatively high conservation in sequence logos, such as in *S*. EAC657, *S*. UW105, and *S*. KORDI-100 (Fig. S5).

As expected, the *procA*/*syncA* genes were distributed across the genomes. Prochlorosins were identified as isolated entities (Fig. 4) or in tandem arrangements (Fig. S6). Group A exhibited the highest number of prochlorosins (113) and predominantly displayed tandem distributions within the GCF (Fig. S6). This GCF comprised up to 16 consecutive precursors within the same cluster. The leader sequences of these specific precursor peptides also showed relatively conserved features, with minor variations in amino acid composition and length. Notably, a homolog of SyncA1, previously associated with SyncM expression (23), was identified and found to be closely related to *S.* UW179A.

### Additional enzymes in prochlorosins BGCs and other neighboring proteins

**Fig 5:**
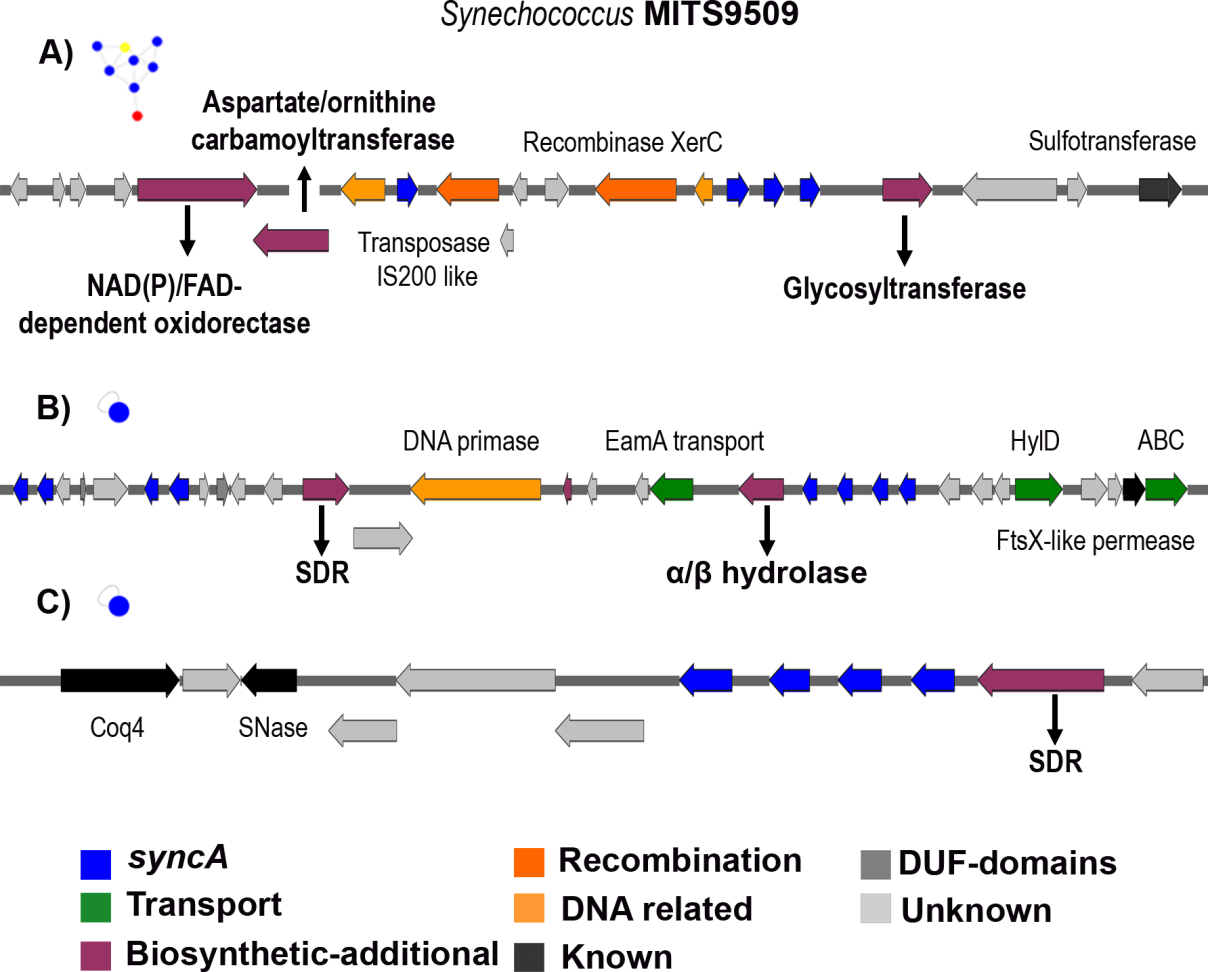
*Synechococcus* MITS9509 Prochlorosin genomic cluster context (Fig. S7). Example of BGC with putative additional annotated biosynthetic enzymes and other neighboring proteins with encoded synechococsins. (**A**) Illustrative instance from GCF group B. (**B**) Singleton BGC is characterized by the presence of *syncA*s (*syncA* genes) and an annotated cluster domain associated with NRP synthetases and PKSs polyketide synthases. (**C**) Singleton BGC featuring *syncA*s and an annotated cluster domain associated with polyketide synthases and a ketoreductase domain. Additionally, this cluster includes Coq4, a protein involved in ubiquinone biosynthesis, an essential component of the ubiquinone biosynthesis pathway. SDR, short-chain oxidoreductase domain.

After identifying all the BGCs encoding *procA*/*syncA* genes, we conducted an analysis of the characteristics of these gene cluster families and singletons within the similarity network. The goal was to explore additional biosynthetic enzymes potentially involved in secondary post-translational modifications of prochlorosin peptides. Among the 81 prochlorosin BGCs, 29 were found to have annotated additional BGCs, as identified by AntiSMASH 5.0. To further characterize these enzymes, we performed BLASTp and Pfam annotations using the HMMER web server from EMBL (https://www.ebi.ac.uk/Tools/hmmer/). In Fig. 5 and Fig. S7, we present examples of the gene cluster landscape of prochlorosin BGCs from *Synechococcus* MIT9509.

One of the most commonly observed encoded enzymes is glycosyltransferases (Fig. 5A) (Glycos_transf_2; pfam00535). While glycosylated RiPPs are rare, they have been described in cacaoidin (34) and glycosins (13). Glycosylation involves the addition of sugar moieties to cysteine, serine, or threonine residues (35). Moreover, some BGCs contain a short-chain oxidoreductase (Fig. 5B) (SDR superfamily: adh_short_C2; pfam13561). Members of this family have been implicated in reducing the N-terminal region to form an N-terminal lactyl moiety in the biosynthesis of the lanthipeptide epilacin (19, 22, 36). Alpha/beta hydrolase families, commonly found in RiPP biosynthetic clusters such as bottromycins and thiostrepton, are also observed (13).

Other families of enzymes detected include NAD(P)/FAD-dependent oxidoreductases, NAD-dependent epimerases/dehydratases (Fig. S7A), various peptidases (S9, M24, M32 families), aspartate/ornithine carbamoyl transferases, SagB-type dehydrogenases (Fig. S7B), methyltransferases, and acetyltransferases. These additional biosynthetic protein families have been identified in previous genome mining studies of RiPP lanthipeptides (7, 19) or mentioned in the context of RiPP biosynthetic clusters (13, 37).

Moreover, within the singleton nodes (Fig. 5B and C; Fig. S7), prochlorosins were found in close proximity to non-ribosomal domains (Fig. 5B) polyketide domains (Fig. 5C) and Type III polyketide synthases (Fig. S7C). It remains uncertain whether these identified enzymes correspond to a single biosynthetic pathway or if they represent two separate clusters with distinct functionalities. These enzymes suggest the possibility of secondary PTMs occurring on prochlorosin/synechococsins or, alternatively, that the genes encoding these enzymes are not directly involved in prochlorosin biosynthesis. This analysis underscores the diversity and shared characteristics of additional biosynthetic enzymes found within the prochlorosin family.

**Fig 6:**
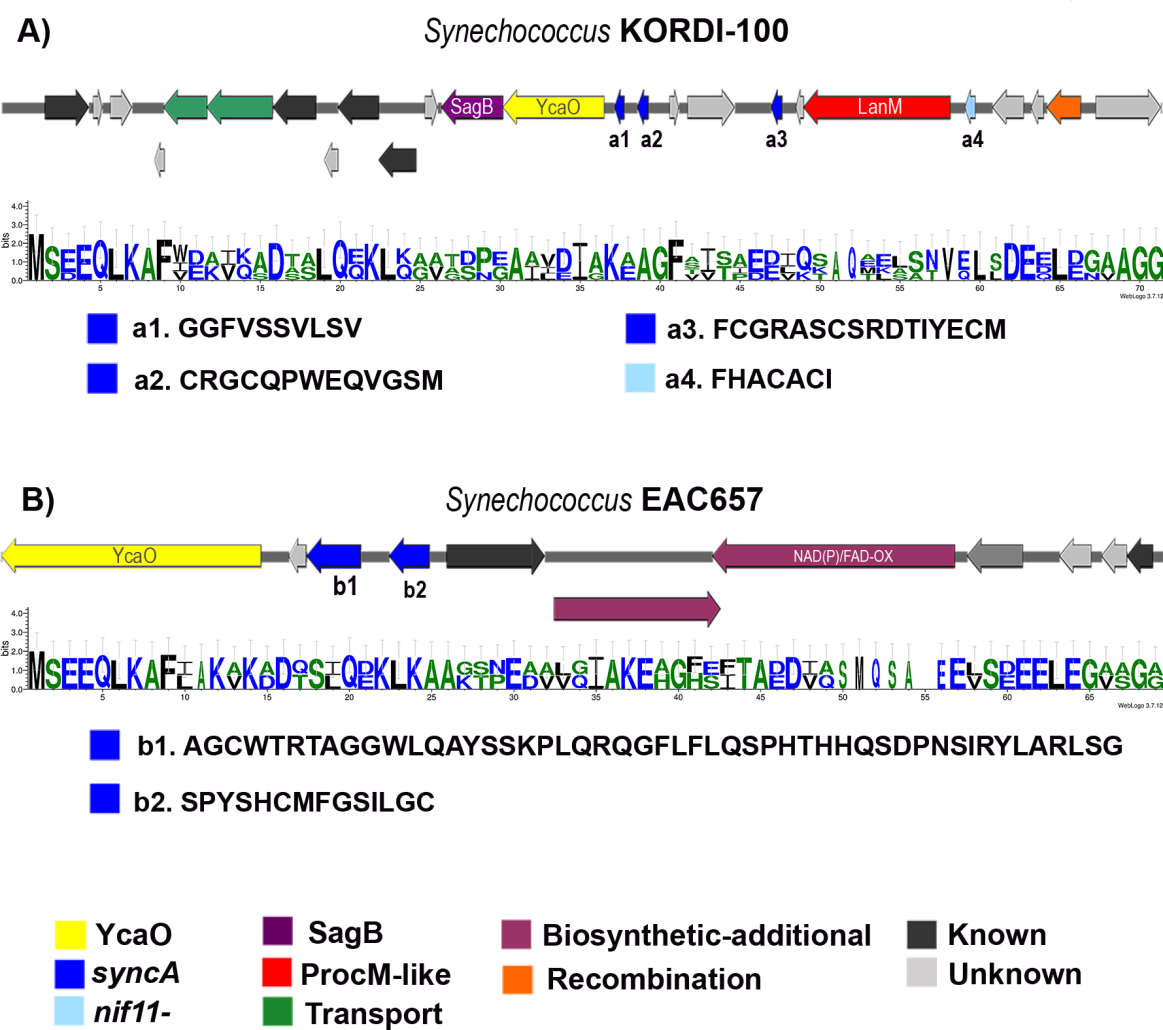
Novel BGCs of a putative LAP/Thiopeptide region in *Synechococcus* strains harboring putative *syncA-* and other *nif11*- precursor family genes. Putative LAP BGC found in this study Putative LAP BGC found in this study of (A) *Synechococcus* KORDI-100 cluster, including a ProcM-like enzyme and (B) *Synechococcus* EAC657. The sequence logo of the conserved leader is shown. Different SyncA putative core sequences are indicated in lowercase.

Finally, we examined the neighboring gene-encoded proteins associated with prochlorosin precursors to identify potential patterns in their distribution. Notably, we observed the presence of proteins involved in various cellular processes, including respiration, photosynthesis, amino acid metabolism, and membrane biogenesis. The presence or absence of specific transport families in certain BGCs suggests the possibility that prochlorosins may be exported without the involvement of dedicated transporters (19). Among the identified transport proteins, ABC sugar transporters, EamA family transporters, resistance ABC efflux proteins, ABC transporters, and ATP-binding proteins were notable. Furthermore, transport-related genes associated with iron and ferric uptake were also observed. Some BGCs exhibited transcriptional regulators such as LuxR or REC_responses, indicating their potential role in regulating the prochlorosin gene cluster (38). Moreover, a variety of recombination-related proteins were identified, including recombinases, site-specific integrases, DNA-breaking and rejoining enzymes, and transposases. The presence of transposase ISO-2000 proteins, previously associated with the prochlorosin family (39) and thiazole/oxazole-modified microcins (TOMM), suggests the potential transfer of genetic clusters among microorganisms (40).

### A newly identified YcaO-containing cluster in the *Synechococcus* genus

The genome mining efforts identified YcaO enzymes (pfam02624) in three distinct BGCs belonging to *Synechococcus* UW179A, *Synechococcus* KORDI-100, and *Synechococcus* EAC567. YcaO proteins are involved in additional post-translational modifications in various families of RiPPs, including azol(in)e-containing peptides (LAP), thiopeptides, bottromycins, and cyanobactins (13, 31). These modifications encompass the formation of azoline heterocycles derived from Cys, Ser, and Thr residues, followed by subsequent oxidation to thiazoles and oxazoles mediated by a dependent dehydrogenase (FMN) (40). The YcaO-mediated modifications produce diverse subclasses of azol(in)e-containing peptides, distinguished by further structural alterations.

The identification of LAP-containing BGCs in *Synechococcus*, which was not previously described in picocyanobacteria (41, 42), prompted us to conduct a BLASTp analysis on the YcaO enzyme identified in *S*. UW179A. As a result, we uncovered three additional marine *Synechococcus* strains, namely, BIOS-E1-4 (CDRI), A15-60 (VII), and A18-25c (VII) (Table S2). Analysis using AntiSMASH 5.0 and BAGEL4 revealed the presence of SyncAs and ProcM-like enzymes in the genomes of these strains. Notably, *S*. BIOS-E1-4 exhibited 2 ProcM-like enzymes, 143 SyncA putative precursors, and 2 YcaO-associated BGCs (Table S2). To facilitate the description of these clusters, we divided them into two subsections for further analysis and characterization.

#### *syncA*-BGC with YcaO and other tailoring enzymes

The first group of BGCs depicted in Fig. 6 and Fig. S8 consists of five BGCs that harbor a *ycaO* gene along with other essential components of the biosynthesis pathways for LAP (SagB), thiopeptides, or lanthipeptides. The ProcM-like BGC in *S*. KORDI-100 includes a ycaO and a *sagB*-like gene. The peptide leader sequences of potential SyncA precursors exhibit a high degree of conservation, while the core sequences vary, with two core substrates lacking Thr and Cys residues. Among these, the core sequence a4. FHACACI bears a resemblance to the cyanobactin ulicylamide (FPTICAC), where both Cys residues are converted to thiazoles (41). Interestingly, *S*. EAC657 shares an identical core sequence with *S*. A15-60 (Fig. S8A) and displays a long SyncA core peptide similar to *S*. A18-25c (Fig. S8B). Furthermore, *S*. A15-60 (Fig. S8A) exhibits a hybrid LAP/thiopeptide structure, as it encodes two putative rSAM enzymes involved in various types of structural modifications in RiPPs, including thiopeptides (13). All the indicated peptide leader sequences belong to Nif11-derived precursors, which have been associated with heterocycles and implicated in the biosynthesis of cyanobactins. However, they have not yet been isolated or characterized (31). The C-terminal region of the prochlorosin leader peptide displays the conserved double Gly-Gly motif, known as NHLP, which is characteristic of other RiPP families such as proteusins or the recently described MpR-RiPP (43). Therefore, these identified PTM enzymes may play a role in secondary modifications within the prochlorosin family, such as the introduction of heterocycles (YcaO) and subsequent oxidation to thiazoles and oxazoles (SagB-like).

#### BGC of a novel LAP/Thiopeptide RiPP in *Synechococcus*

**Fig 7:**
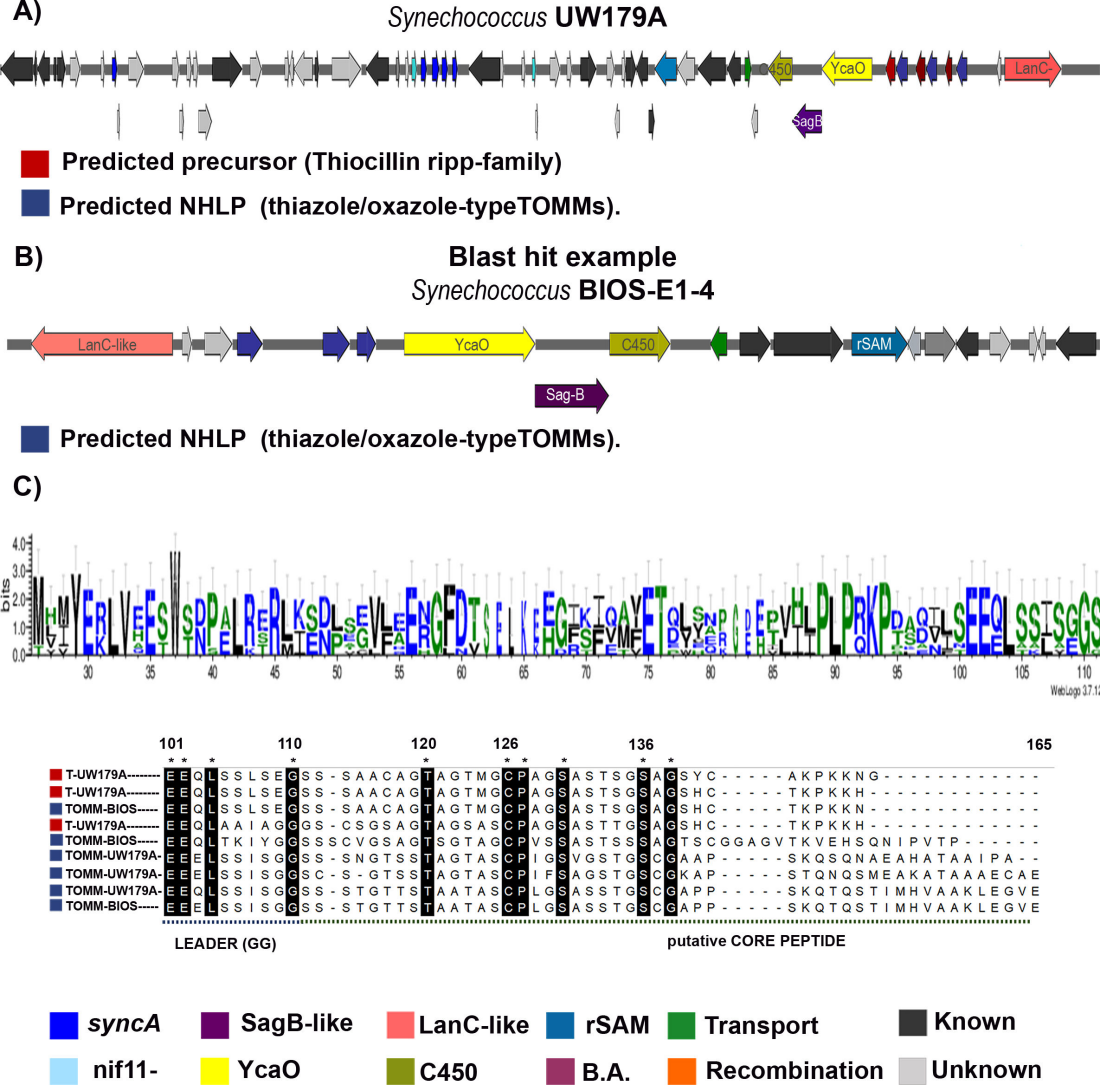
Homologous BGC genomic regions containing YcaO in two marine *Synechococcus* strains. (**A**) *Synechococcus* UW179A BGC organization. Two RiPP families of putative precursor are indicated. Thiollicin family (red square) and TOMM/NHLP family (dark blue square). (**B**) *Synechococcus* BIOS-E1-4 BGC. Putative precursor TOMM/NHLP (dark blue square). (C) Sequence logo of all precursors showed a highly conserved leader sequence including the double Gly-Gly cleavage motif Alignment of the C-terminal portion of precursor sequence from Thiocillin and TOMM/NHLP family. Leader and core sequence are indicated. Thiocillin-like conserved C-terminal is indicated (A/T-KPKKH-N/H). Conserved regions are marked in black and asterisk.

The second set of YcaO clusters originates from the strains *S*. UW179A and *S*. BIOS-E4-1, as shown in Fig. 7A and B. These two BGCs exhibit homology and contain two additional PTM enzymes annotated by AntiSMASH and nine putative precursor genes that are not ProcA/SyncA. Initially, we conducted a BLASTp analysis for each of the novel precursors. Three precursors were annotated as thiocillin RiPPs (Fig. 7A), while the remaining ones were annotated as TOMM/NHLP (Fig. 7A). This precursor-sequence similarity lies within the substrate’s leader section. This leader family can also be found in BGCs responsible for cyanobactin production (40). However, these described BGCs lack the presence of a PatA/PatG-like protease, which is characteristic of the cyanobactin group. As a result, this particular group, including the Nif11 family, has not been assigned to any known RiPP class or subclass (31). It is worth noting that no similar precursor peptides were identified in the BLASTp search or previous studies.

A sequence similarity logo of the putative precursor leader (Fig. 7C) reveals similarities to the recently described leader of MprE, which possesses a Pro-rich region, a YcaO, and the GG motif (43). These peptides from *Methylovulum psychrotolerans* Sph1 are modified by a promiscuous YcaO cyclodehydratase (43). The alignment of the C-terminal core region illustrates that the peptides found in these clusters are enriched in heterocyclizable residues such as Cys, Ser, and Thr. Specific residues are conserved at particular positions (Fig. 7C, precursor alignment), including Thr120, Cys126, Pro127, Ser130, Ser136, and Gly137. Two groups of core precursors can be distinguished. The thiocillin-like core peptides (Fig. 7C, red) are shorter and share a common A/T-KPKKH-N/H C-terminal motif. In contrast, the NHLP group exhibits an extended core. It remains unclear whether these core peptides will undergo processing and cyclization similar to cyanobactins if the long core undergoes proteolysis to release a smaller bioactive core (44) or if they may represent linearly modified LAP/RiPPs (31).

To find clues about the type of structural modifications these peptides could undergo, we performed a deeper analysis of these unique clusters using homology modeling and analysis of the sequence alignment of the annotated PTM enzymes (Fig. S9). First, the YcaO protein had homology with the cyanobactin cyclodehydratase LynD and TruD (Fig. S9A). These conserved ATP- Mg^+2^-dependent YcaO will heterocyclize cysteine residues into thiazolines in patellamide-like products; this reaction is presumably the initial step in the processing (28, 44). In the alignment, we identified the conserved ATP-binding domain described for the YcaO superfamily (45) and LynD (28) (Fig. S9A). Thus, the identified YcaO could lead to the heterocyclization of Cys residues (conserved Cys_126_). The model of the SagB-like sequence showed similarity to the FMN-dependent Thc_ox_ (46) from the cyanothecamide biosynthetic pathway (Fig. S9B). This enzyme can convert thiazolines to thiazoles (44, 46). The oxidase domain can be found fused to YcaO or, in this case, standalone. The FMN-binding motif (47) was identified (Fig. S9B).

Furthermore, two more modifications could be installed. The BGC has an annotated Cytochrome 450 (Fig. 7A and B). The crystal structure of C450 involved in the synthesis of RiPPs has yet to be elucidated. Hence, no modeling was performed. Nevertheless, these tailoring enzymes are part of thiopeptide pathways and are known to catalyze hydroxylation and epoxidation reactions (22). A specific example is the thiocillin-like molecule called nocardithiocin, for which hydroxylation of a dehydroalanine (Dha) (30) has been proposed.

Finally, AntiSMASH annotated a putative LanC-like enzyme (Fig. 7A and B). The predicted model of this LanC-like_UW179A_ enzyme (Fig. S10) showed homology with class II LanM enzymes, specifically LicM2 and CylM, as confirmed by BLASTp analysis. Upon close examination of the dehydration domain in the model (Fig. S10C and D), we identified the active site described in previous studies for CylM (22), with only one difference: CylM-H349 − LanC-W281. In line with this observation, the alignment of LanC-like_UW179A_ with CylM occurred in the dehydration domain (Fig. S10E and D). Therefore, we suggest that this putative PTM enzyme is most likely a LanB-like dehydratase. If a modification occurs, it will most likely involve a dehydration step.

We built a phylogenetic tree with each enzyme of the described biosynthetic pathway (Figures S11, S12 and Table S3). The YcaO protein sequence logo showed the conserved YcaO domain discussed above. (Fig. S11). The phylogenetic tree (Fig. S11) has two main clades, one with thiopeptide and LAP families and a second with YcaO enzymes from the cyanobactin, goadsporin, and *Synechococcus* genera. Interestingly, prochlorosin-containing YcaO enzymes are closely related to cyanobactin enzymes. However, they are grouped into separate branches. *Synechococcus* strains with thiocillin and TOMM/NHLP precursors diverted to a secondary branch of this clade, meaning they are more distantly related to the cyanobactin family. The same pattern was observed in the SagB-like tree (Fig. S12A). Finally, for the LanC-like’s, LanB-like’s, based on our homology analysis, are clustered in their own branch, distant to the ProcM-like group and LanM. The NisB dehydratase occupied a separate branch outside these two groups (Fig. S12B).

Taking together the biosynthetic gene cluster and phylogenetic tree analysis, we suspect it is feasible that either prochlorosins or newly identified precursor substrates may undergo at least two modifications, including the heterocyclization of Cys/Ser/Thr (most probably a Cys to thiazolines) and the oxidation of the azoline to azole heterocycles. The second set of substrates can include two more structural changes. The dehydration of Ser / Thr (LanB-like) and subsequent hydroxylation (C450). We suggest this cluster can result in a new modified hybrid azol(in)e-containing RiPPs. However, we cannot define which class of RiPP it might belong to because the BGC contains enzymes from the cyanobactin, LAP, and thiopeptide pathways.

## DISCUSSION

**Fig 8:**
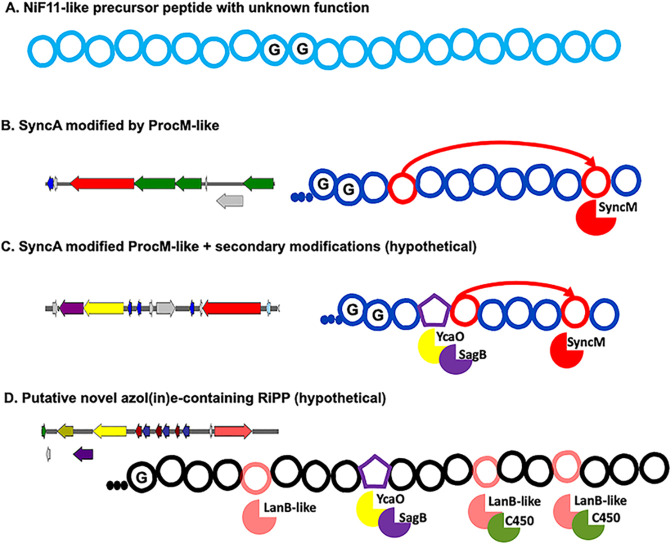
Examples of RiPP putative precursor peptides describe in this study. (**A**) The Nif11 precursor peptide is highly conserved among the studied prochlorosins. (**B**) Precursor peptides of Prochlorosins (ProcA/SyncA) that undergo modification by ProcM-like enzymes (ProcM or SyncM) and possess a lanthionine ring highlighted in red. (**C**) Hypothetical secondary modifications of SyncA precursor peptides facilitated by neighboring PTM enzymes: YcaO (heterocyclization of Cys) and SagB-like (oxidation to azole heterocycles). (**D**) Hypothetical putative azol(in)e-containing RiPP, involving enzymes such as YcaO (heterocyclization of Thr or Cys), SagB-like (oxidation of heterocycles), LanB-like (dehydration of Ser to Dha), and C450 (hydroxylation of Dha).

Cyanobacteria are rich in bioactive secondary metabolites, characterized by their diverse structures originating from complex biosynthetic gene clusters (5, 6). This study specifically focuses on the bioinformatic analysis of the prochlorosin lanthipeptides belonging to the Nif11 family, which are a remarkable illustration of combinatorial biosynthesis (2, 8, 48). Additionally, we provide an extensive description of the genomic context surrounding this lanthipeptide family, along with the identification of a novel putative RiPP. A comprehensive overview of the intriguing peptides discovered in this study is presented in Fig. 8.

We employed a network similarity program to investigate the distribution of prochlorosin biosynthetic clusters and identify similarities among gene clusters encoding *procA*/*syncA* genes. Surprisingly, our analysis revealed the presence of different enzymes involved in post-translational modifications within the prochlorosin region. While some of these enzymes are already known in various RiPP biosynthetic pathways, others have unknown functions. It remains to be explored whether prochlorosins undergo secondary modifications although we propose that this is highly likely. An interesting example is the presence of SyncAs within YcaO clusters, which could potentially lead to the formation of azole heterocycles (Fig. 6 and 8C). This observation raises the question of whether some prochlorosins require secondary modifications to be biologically active. Furthermore, the unusually long leader peptide (60 amino acids) found in this family may contain additional binding motifs. It has been demonstrated that ProcM only requires about three-quarters of the ProcA leader, suggesting that the remaining portion of the leader peptide could serve a different role. Additionally, the NHLP/Nif11 double Gly-Gly motif, shared with other RiPP families, may be recognized by other post-translational modification enzymes.

The biological function of the prochlorosin family remains a mystery. We examined the genomic context surrounding prochlorosins to hypothesized potential biological activities and gain further insight. However, the analysis revealed a complex genomic landscape with a diverse array of encoded proteins. This peptide diversity suggests that ProcA/SyncA precursors may have different functions. The presence of sensor kinases, transcriptional response regulators, and repressors in some gene clusters suggests that environmental changes or the presence of other microorganisms may regulate their expression. Hypothesizing about functionality is challenging, as these peptides may serve various roles, including signaling, quorum sensing, and antimicrobial activity, among others. A transcriptional analysis of *Prochlorococcus* MIT9313, which possesses 29 diverse prochlorosins, demonstrated changes in expression upon exposure to a marine heterotrophic bacterium (49). Moreover, it has also been suggested that prochlorosins could serve as a valuable nitrogen source and contribute to sulfur reduction (50).

Secondary metabolites play crucial roles in the survival of marine microbes, acting as defense mechanisms, attractants, deterrents, or signaling molecules to mediate interactions with other organisms (11, 51). The production of these metabolites incurs a high metabolic cost (51), indicating that the maintenance and evolution of the prochlorosin lanthipeptide family must confer advantages to picocyanobacteria populations. Importantly, genome diversification is essential for niche adaptation (52). The maintenance and diversity of the prochlorosin trait may involve two evolutionary mechanisms: (i) horizontal gene transfer, as suggested by the presence of recombination-related proteins in multiple gene clusters and (ii) structural diversity resulting from insertion-deletion events in the prochlorosin core sequence, which is constantly subjected to selective environmental pressures (3, 40, 51).

The discovery of a new group of gene clusters containing YcaO enzymes in the genus *Synechococcus* was an unexpected finding. Interestingly, cluster analysis revealed that the identified LanC-like enzyme, as annotated by AntiSMASH, exhibited a domain similar to LanB-like enzymes. Dehydration plays a crucial role in the biosynthesis of thiopeptides (22) and LAPs like goadsporin (53). Dehydroamino acids are introduced in these pathways by a split dehydratase, which shares similarities with class I LanB enzymes (22). The separated genes in the goadsporin cluster showed partial homology with the N-terminal glutamylation and C-terminal elimination domains of LanB dehydratases (22, 53). However, the LanB-like structure observed in the identified gene cluster appears to be more closely related to class II LanM enzymes, where the dehydration reaction occurs via phosphorylation (22). Therefore, this LanB-like enzyme may differ from previously described lanthipeptide dehydratases involved in azol(in)e-containing RiPP biosynthetic pathways. A comparable example can be found in the recently discovered diphosphorylated RiPP phospeptin, where an annotated LanC-like enzyme displayed similarity to the dehydratase domain of CylM (9, 54). However, unlike the findings of this study, the characterized modification enzyme lacks the critical active site residues required for phosphate elimination, resulting in two phosphorylated Thr residues (9). Based on this, we hypothesize that the identified precursors in our study may give rise to a novel azol(in)e-containing RiPP with an additional dehydration step (Fig. 7 and 8D). Members of this group are known to exhibit antimicrobial properties similar to thiocillin (55), thiostrepton A, thiomuracin, planazolicin, and others (56).

### Conclusion

This study provides insights into the genomic landscape of the highly variable prochlorosin family, which is found in *Synechococcus* and *Prochlorococcus* strains. Despite their widespread distribution, the functional roles of prochlorosins remain unknown and are likely to be diverse. In this study, we have expanded the repertoire of prochlorosins by identifying several new unique peptides. Additionally, the presence of tailoring enzymes and the characteristic large leader peptide suggest that some prochlorosins may undergo additional modifications. These modifications could involve the conversion of non-dehydrated Ser/Thr or Cys residues into azole and azoline heterocycles. Notably, this study also unveils a novel group of gene clusters encoding various components of azol(in)e-containing RiPP biosynthetic pathways in the *Synechococcus* genus. These findings highlight the potential for the synthesis of bioactive compounds with azole and azoline modifications in *Synechococcus* species. Further investigation is required to elucidate the precise functions and biological activities of prochlorosins and their modified forms.

## MATERIALS AND METHODS

### Genome sequence and phylogenetic analysis

We performed a genome-scale comparison of the 47 marine *Synechococcus* and *Prochlorococcus* strains. The genome sequences were obtained from NCBI in 2019, and the corresponding accession numbers can be found in Table S1. The comparison was carried out using GTDB-Tk v1.5.0 (57) with the default parameters, using the GTDB r202 release as the reference database. A multiple sequence alignment of the 117 identified markers from the 47 genomes was generated. Subsequently, a maximum likelihood phylogenetic tree was constructed using IQ-TREE multicore version 1.6.12 (58). The best-fit substitution models LG + R5 were selected by ModelFinder (59) and 1,000 ultra-fast bootstraps were performed to assess the tree’s robustness. For tree visualization, we utilized the ggtree R package (60).

### Mining for prochlorosin lanthipeptide family and RiPPs

We conducted genome mining using the AntiSMASH 5.0 platform (26) available at https://antismash.secondarymetabolites.org/#!/start. Each genome was uploaded, and the platform was used to identify biosynthetic gene cluster (BGC) regions. Subsequently, the identified clusters were further analyzed using the protein BLASTp, accessible at https://blast.ncbi.nlm.nih.gov/Blast.cgi. For subsequent analyses, we focused on BGCs containing prochlorosins, the Nif11-like family, and novel RiPPs. To compare the putative substrates of the *procA/syncA* genes identified by AntiSMASH, we performed an additional analysis using the BAGEL4 platform (33) available at http://bagel4.molgenrug.nl. Finally, we BLASTp each ProcA/SyncA-containing genomes to further manually curate the prochlorosin/synechococsin database and identify putative precursors encoded in the different genomes. This allowed us to gain further insights into the potential functions and characteristics of the identified ProcA/SyncA substrates.

### Similarity network analysis of identified BGCs

We utilized the BiG-SCAPE (27) platform (https://git.wageningenur.nl/medema-group/BiG-SCAPE) to generate a similarity network based on the annotated AntiSMASH files. This network allowed us to explore the diversity of the identified biosynthetic gene clusters (BGCs). Subsequently, we employed Cytoscape v3.7.0 (http://www.cytoscape.org/) to visualize the distance matrix generated by BiG-SCAPE. Default parameters were used for both software tools, ensuring consistency and standardization in the analysis process.

### Sequence logo of putative prochlorosins amino acid sequence and PTM enzymes

To validate the protein sequences and accession numbers obtained from the identified putative biosynthetic modification enzymes and precursor peptides in the BGC, we performed a BLAST search (61) using the amino acid sequences. This search was conducted on the NCBI web service to confirm their identities and retrieve relevant information. Additionally, we conducted Pfam annotation of the PTM enzymes of interest using the HMMER web server from EMBL (https://www.ebi.ac.uk/Tools/hmmer/). This allowed us to identify specific protein domains and gain insights into their functional characteristics. To analyze the alignment of the putative peptides and post-translational modification enzymes, we utilized the ClustalW algorithm within MEGA11 (62). This alignment facilitated the comparison of sequence similarities and differences among the families of interest. Furthermore, we employed the WEB-LOGO server (63) to generate sequence logos based on the aligned precursor peptides and enzymes. These sequence logos visually represented conserved motifs within each family, providing valuable information about their shared characteristics and potential functional elements.

### Modeling by homology and phylogenetic analysis of the modification enzymes encoded in the YcaO-biosynthetic gene cluster

The ExpAsy SWISS-MODEL web server (64 – 70), available at https://swissmodel.expasy.org, was utilized for modeling and aligning the various tailoring enzymes within the BGC of interest. This platform allowed us to generate structural models and alignments of the enzymes, aiding in the understanding of their three-dimensional structures and potential functional characteristics. For the phylogenetic tree analysis, we employed MEGA X (62), a software tool specifically designed for molecular evolutionary analysis. The evolutionary history was inferred using the Neighbor-Joining method (71), which constructs a tree based on the pairwise distances between sequences. The tree was drawn to scale, with the branch lengths representing the evolutionary distances computed using the Poisson correction method (72). Default parameters were applied throughout the analysis to ensure consistency and comparability.

## SUPPLEMENTAL MATERIAL

Supplemental Tables and Figures spectrum.03611-23-s0001.pdf Tables S1 to S3 and Figures S1 to S12.

ASM does not own the copyrights to Supplemental Material that may be linked to, or accessed through, an article. The authors have granted ASM a non-exclusive, world-wide license to publish the Supplemental Material files. Please contact the corresponding author directly for reuse.
