# Supplementary material for: Uncovering the diversity and distribution of biosynthetic gene clusters of prochlorosins and other putative RiPPs in marine Synechococcus strains
Source: Microbiol Spectr. 2023 Dec 13;12(1):e03611-23. doi: 10.1128/spectrum.03611-23 (PMC10783134; doi:10.1128/spectrum.03611-23)
Supplement: Supplemental Tables and Figures — Tables S1 to S3 and Figures S1 to S12. [file spectrum.03611-23-s0001.pdf]

Uncovering the diversity and distribution of biosynthetic gene clusters of prochlorosins and other putative RiPPs in marine *Synechococcus*

Patricia Arias-Orozco,[a] Lu Zhou,[a,b] Yunhai Yi,[a] Rubén Cebrián,[a,c,d] Oscar P. Kuipers[a#]

[a] Department of Molecular Genetics, University of Groningen, Groningen, Nijenborgh 7, 9747AG, Groningen, The Netherlands.

[b] Department of Plants and Crops, Faculty of Bioscience Engineering, Ghent, University, Belgium.

[c] Department of Clinical Microbiology, Instituto de Investigación Biosanitaria ibs.GRANADA, San Cecilio University Hospital, Av. De la Innovación s/n, 18016, Granada, Spain.

[d] CIBER de Enfermedades Infecciosas, CIBERINFEC, ISCIII, Madrid, Spain

SUPPORT INFORMATION

TABLES AND FIGURES

Table S1. ***Synechococcus* and *Prochlorococcus* strains used in genome mining study.** Accession number, Predicted BGC's with putative RiPPs, BGC's with putative procA genes, identified prochlorosins in this study and referenced prochlorosins. Cluster with the LanM mentioned in this study and ID accession number.

GROUP I	ACCESSION	REF. CLADE	REFERENCE	PREDICTED RIPPS BGC	BGCS *proca/synca*	PUTATIVE *proca/synca*	REF. *proca/synca*	LanM CLUSTER	ID ACC. ProcM-like	YCAO CLUSTER	ID ACC. YcaO	ID ACC. C450	ID ACC. SagB
***P.* MIT9313**	GCF_000011485.1	LLIV	*(1)*	13	7	40	29	NC_005071.1.region003	CAE20425.1				
***P.* MIT9515**	GCF_000015665.1	HLI	*(1)*	2									
***P.* MED105**	GCA_002692385.1		Tara Oceans Consortium	1									
***P.* CCMP1986**	GCF_000011465.1		DOE Joint Genome Institute	0									
***P.* AS9601**	GCF_000015645.1	HLII	*(1)*	1									
***P.* MIT9312**	GCF_000012645.1	HLII	*(1)*	1									
***P.* MED630**	GCA_002689785.1		Tara Oceans Consortium	0									
***P.* SP3043**	GCA_002720205.1	HLI	Tara Oceans Consortium	0									
***P.* NATL2A**	GCF_000012465.1	LLI	*(1)*	2									
***P.* MED4**		HLI	*(1)*	1									

**GROUP II**													
***S.* RS9916**	GCF_000153825.1	IX	*(1)*	13	6	20	19	NZ_DS022299.1.region011	WP_007098833.1				
***S.* UW105**	GCF_900473935.1	XVI	*(2)*	17	7	16	11	NZ_UCNN01000030.region001	WP_115121540.1				
***S.* UW140**	GCF_900474295.1	XVI	*(2)*	9	1	2	1		WP_295536282 (NCBI: NZ_CAMDUZ010000129.1)				
***S.* NP17**	GCA_002729835.1	N/A	Tara Oceans Consortium	7									
***S.* CC9311**	GCF_000014585.1	I	*(1)*	8									
***S.* WH 8020**	GCF_001040845.1	I	*(1)*	7									
***S.* WH 8016**	GCF_000230675.1	I	*(1)*	8	1	1	1	NZ_AGIK01000002.region001	WP_006853651.1				
***S.* UW179B**	GCF_900474245.1	I	*(2)*	3									
***S.* WH 7803**	GCF_000063505.1	V	*(1)*	10	1	1	1						
***S.* WH 7805**	GCF_000153285.1	VI	*(1)*	5									
***S.* EAC657**	GCA_002693285.1	N/A	*(1)*	11	8	23	20	NZPI01000042.1	MAN20183.1		MAN20207.1		
***S.* MIT S9509**	GCF_001631935.1	CRDI	*(2)*	30	21	83	79	NZ_LVHV01000019.region001	WP_082823450.1				

***S.* UW179A**	GCF_900473965.1	CRDI	*(2)*	27	19	79	53	NZ_UCNJ01000025.region005	WP_114994184.1		WP_170953582.1		WP_114992948.1
								WP_114992952.1 (LanC-like)					
***S.* CPC100**	GCA_002685515.1	N/A	Tara Oceans Consortium	3	1	2							
***S.* MIT S9508**	GCF_001632165.1	CRDI	*(1)*	9	4	8	8	NZ_LVHU01000004.region001	WP_067094036.1				
***S.* GEYO**	GCF_900473955.1	CRDI	*(2)*	5									
**GROUP III**													
***S.* RCC307**	GCF_000063525.1	X	*(2)*	5									
***S.* KORDI-49**	GCF_000737575.1	WPC1	*(1)*	4									
***S.* MED850**	GCA_002700765.1	N/A	Tara Oceans Consortium	0									
***S.* KORDI-100**	GCF_000737535.1	UC-A	*(1)*	12	5	10	10	NZ_CP006269.1.region009	WP_051847417.1		WP_038545038.1		WP_051847413.1
***S.* CC9616**	NZ_KI911558.1	XX	*(2)*	3									
***S.* ARS1019**	GCA_002690325.1	N/A	Tara Oceans Consortium	7									
***S.* SAT82**	GCA_002706715.1	N/A	Tara Oceans Consortium	1									

***S.* WH 8102**	GCF_000195975.1	III	Tara Oceans Consortium	5									
***S.* CPC35**	GCA_002684175.1		Tara Oceans Consortium	3									
***S.* CC9902**	GCF_000012505.1	IV	Tara Oceans Consortium	8									
***S.* BL107**	GCF_000153805	IV	*(1)*	6									
***S.* MED650**	GCA_002691345.1		Tara Oceans Consortium	1									
***S.* UW106**	GCF_900474015.1	XV	*(2)*	4									
***S.* KORDI-52**	GCF_000737595.1	WPC2	*(2)*	6									
***S.* CC9605**	GCF_000012625.1	II	Cubilloz-Ruiz et al 2017	5									
***S.* N5**	GCF_900473925.1	II	*(2)*	3									
***S.* N26**	GCF_900473975.1	II	[2]	4									
***S.* RS344**	GCA_002724845.1		Tara Oceans Consortium	2									
***S.* N19**	GCF_900474045.1	II	*(2)*	4									
***S.* UW86**	GCF_900474085.1	II	*(2)*	3									
***S.* WH 8109**	GCF_000161795.2	II	*(2)*	4									

***S.* N32**	GCF_900473895.1	II	*(2)*	4									
**Total**				**287**	**81**	**285**	**232**						

Table S2. *Synechococcus* strains identified by BLAST in analysis of the YcaO cluster

Blast identified	ACCESSION	REF. CLADE	REFERENCE	PREDICTED RIPPS BGC	PUTATIVE *proca/synca*	ProcM-like CLUSTER	ID ACC. ProcM/LanC-like	YcaO CLUSTER	ID ACC. YcaO	ID ACC. C450	ID ACC. SagB
***S.* BIOS-E1-4**	NZ_CP047935.1	CDRI	*(3)*	9	143	CP047935.1.region008	QNI55536.1				
						CP047935.1.region001	WP_186539462.1	CP047935.1.region004	QNI54268.1		WP_186540155.1
						CP047935.1.region005 **(LanC-like)**	WP_186540444.1	CP047935.1.region005	WP_186540456.1	WP_186540460.1	WP_186540458.1
***S.* A15-60**	NZ_CP047933	VII	*(3)*	6	24	NZ_CP047933.1.region004	WP_186500453.1		WP_186501049		WP_186501048.1
***S.* A18-25c**	NZ_CP047957	VII	*(3)*	4	32	NZ_CP047957.1.region003	WP_222929995.1		QNJ18681.1		WP_186470014.1
Total					199						

Table S3. RiPPs tailoring enzymes (PTM) ID accession for phylogenetic tree analysis.

Organisms	ID YcaO	Enzyme	RiPP subfamily	RiPP family
Enterobacteriaceae	WP_001526802.1	YcaO	Microcin	LAP
Burkholderia pseudomallei	EIF80112.1	YcaO	NHLP precursor	
Bacillus toyonensis	WP_000512733.1	YcaO	Hakacin	LAP
Bacillus	WP_015239353.1	YcaO	Plantazolicin	LAP
Bacillus cereus	WP_011110463.1	YcaO	Thiocillin	Thiopeptide
Streptomyces hygroscopicus	ACS50130.1	YcaO	cyclothiazomycin	Thiopeptide
Streptomyces laurentii	ACN52298.1	YcaO/TrsH	/Thiostrepton	Thiopeptide
Streptomyces actuosus	WP_110629801.1	YcaO	Noshipeptide	Thiopeptide
Streptomyces sp. WMMB 272	AFU90403.1	YcaO		Bottromycin D
Oscillatoria nigro-viridis/	WP_015177263	YcaO	Viridisamide A	Cyanobactin
Lyngbya aestuarii	4v1t.1 (PDB)	YcaO/LynD	Aestuaramide	Cyanobactin
Prochloron didemni	AAY21153.1	YcaO/PatD	Patellamide	Cyanobactin
Nostoc spongiaeforme	ACA04483.1	YcaO/TenD	Tenuecyclamide	Cyanobactin
Prochloron sp	ACA04490.1	YcaO/TruD	Trunkamide	Cyanobactin
Amycolatopsis sp.	ATY16955.1	YcaO	Goadsporin	LAP
Cyanothece sp. PCC 7425	5LQ4 (PDB)	SagB/ThcOX		Cyanobactin
Staphylococcus aureus	WP_000611635.1	SagB		
Enterococcus faecalis	AAK67266.1	LanM/CylM	Class II	Lanthipeptide

Streptococcus sp. 3.1	WP_201043397.1	LanM	Class II	Lanthipeptide
Bacillus thuringiensis	WP_087995090.1	LanM	Class II	Lanthipeptide
Lactobacillus melliventris	WP_110445472.1	LanM	Class II	Lanthipeptide
Bacillus licheniformis	ADW08735.1	LanM/LicM2	Class II	Lanthipeptide
Lactococcus lactis	CAA48381.1	NisB	Class I	Lanthipeptide
Streptomyces sp. TP-A0584	BAE46922.2	Lant_dehydr_C (goadsporin biosynthetic protein)	Goadsporin	LAP
Streptomyces sp. TP-A0584	BAE46921.1	Lant_dehydr_N (goadsporin biosynthetic protein)	Goadsporin	LAP

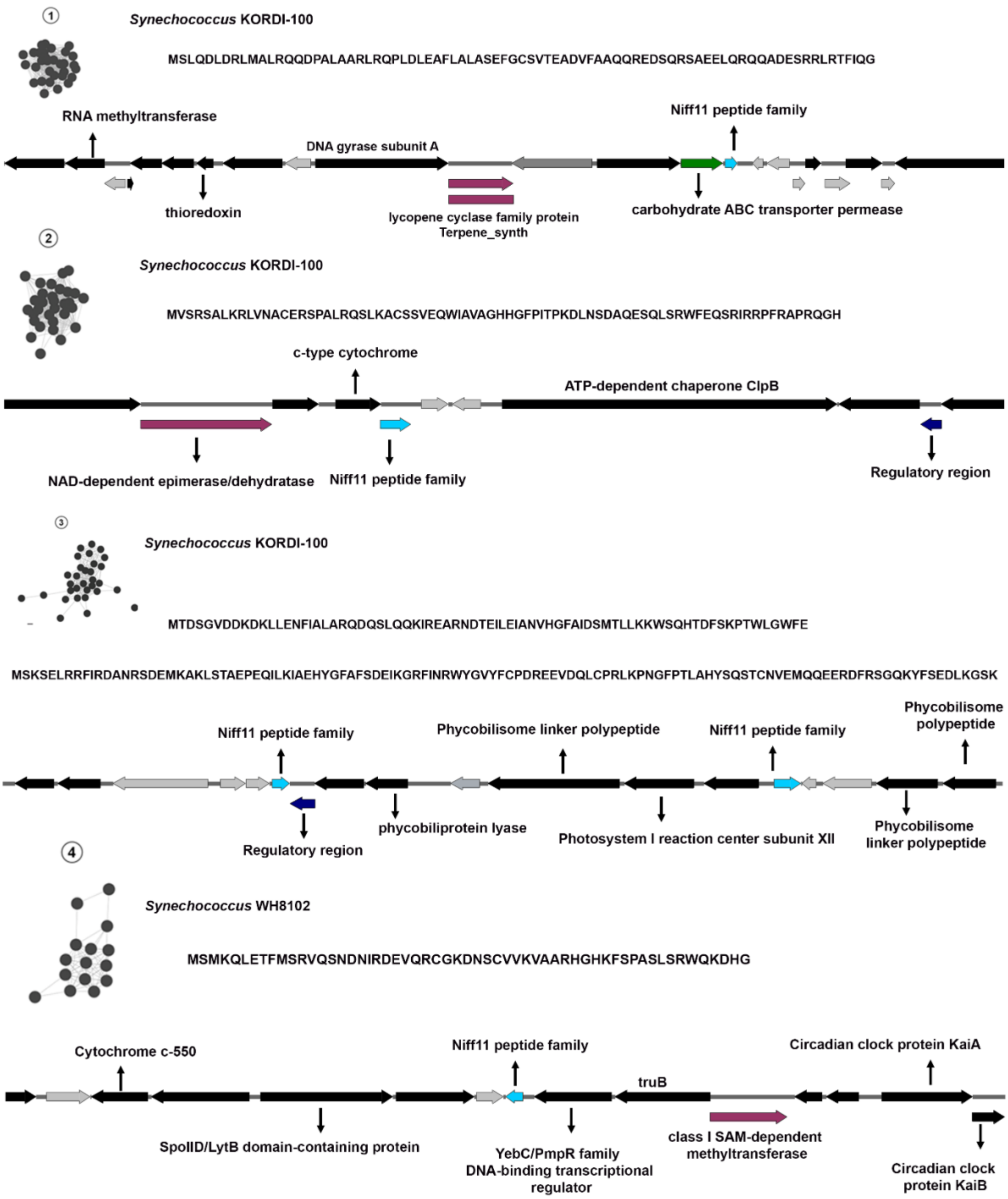

Figure S1. Example of putative BGC's gene cluster families 1 to 4 containing differently conserved ribosomally synthesized peptide precursors from the Nif11 family (Light blue). Sequences of the identified precursor are shown. As BGC boundaries are unknown, the putative encoded tailoring enzymes may be part or not of the BGC modification machinery.

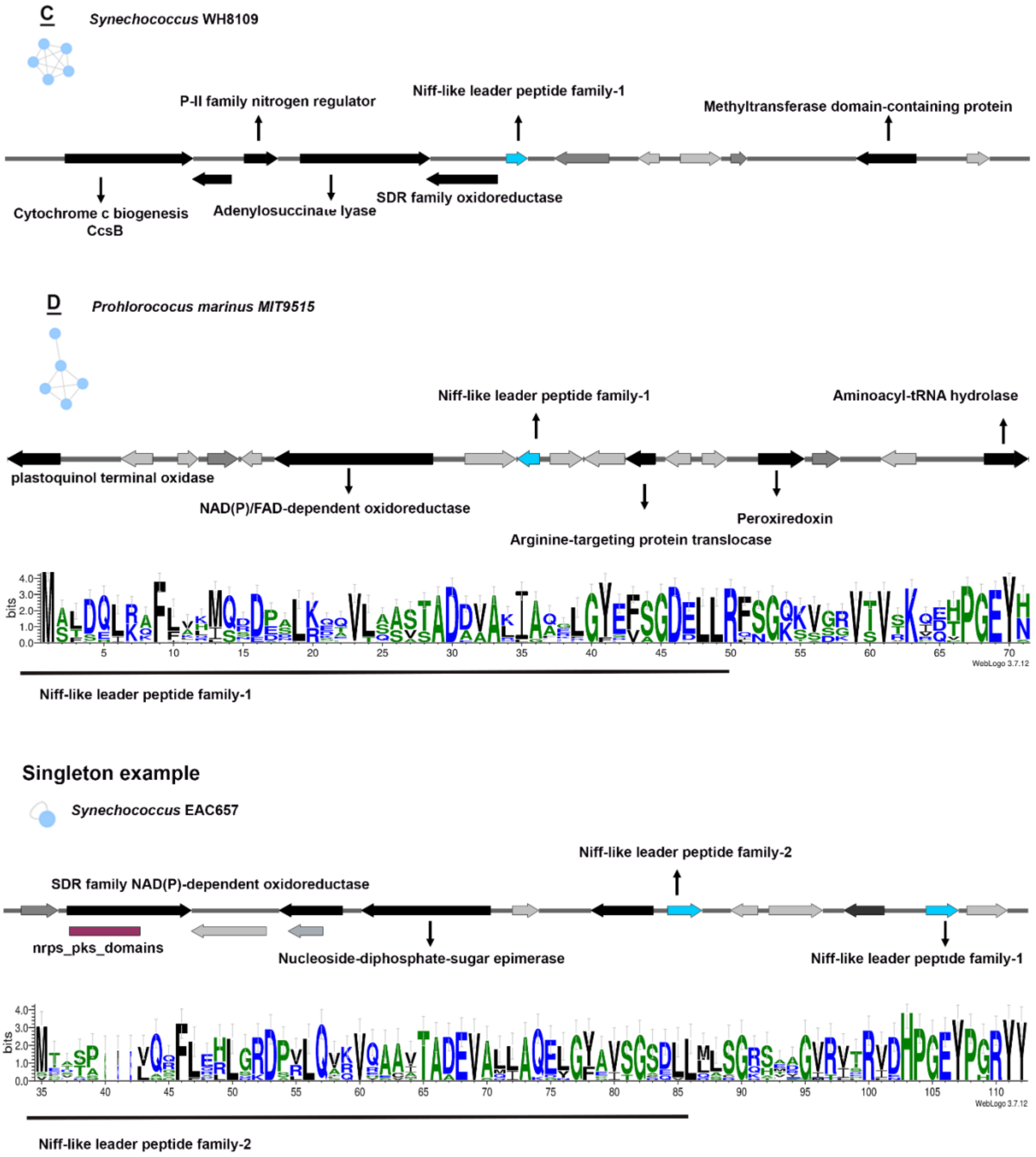

Figure S2. Example of putative BGCs in gene cluster families C to D and one single node containing one or two conserved ribosomally synthesized peptide precursors from the Nif11 family (light blue). Sequence logo (4) of the conserved identified precursor is depicted. As BGC boundaries are unknown, the putative encoded tailoring enzymes may be part or not of the BGC modification machinery.

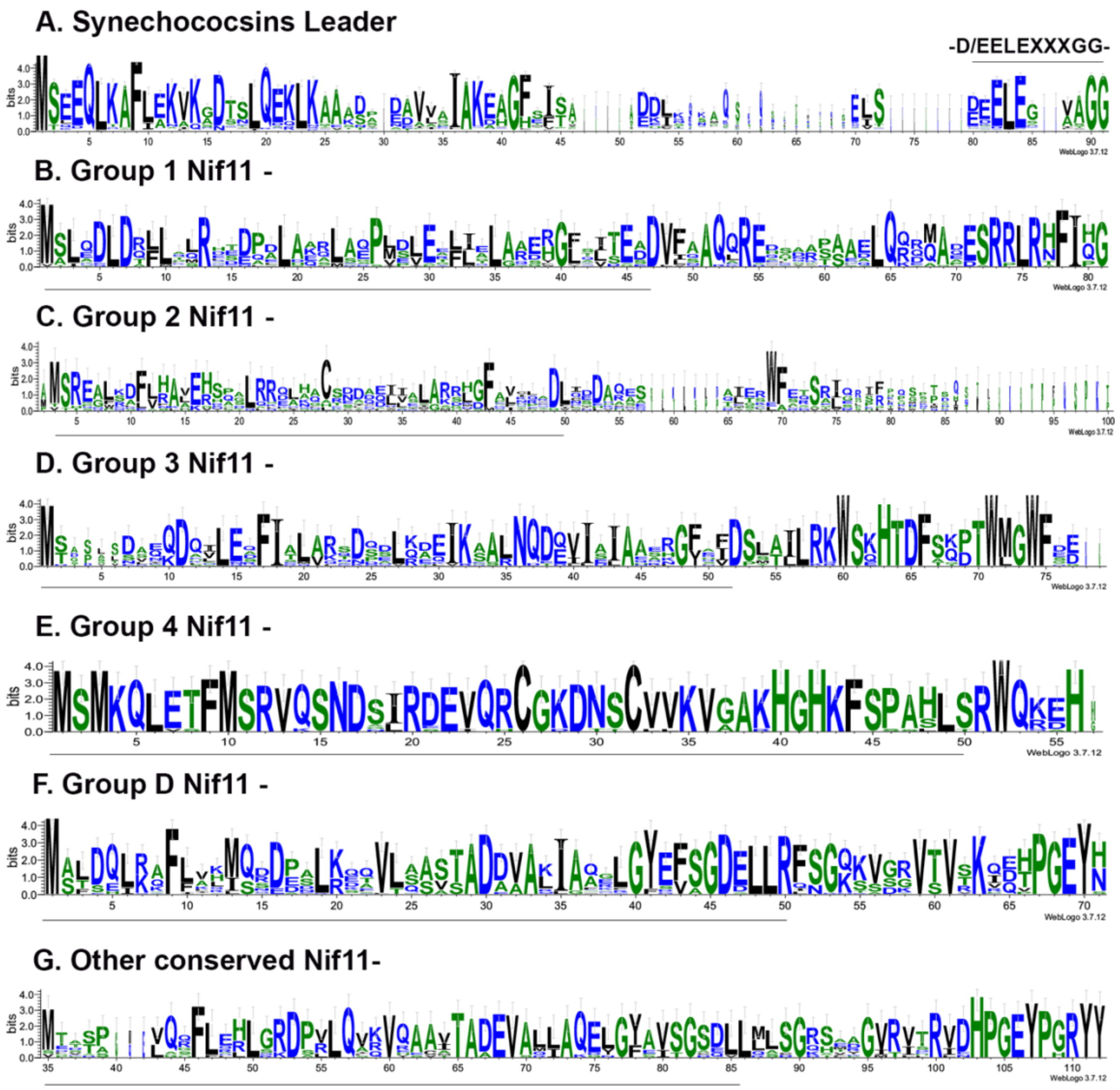

Figure S3. Examples of putative different Nif11- precursors. Sequence logo (4) identified Nif11- family precursors identified with the genome mining analysis. Synechococsins leader has a N-terminal (-MSEEQL-) and the characteristic C- terminal motif (-D/EELExxxGG-). Lines under the peptides indicated putative region of Nif11- like leader peptides identified in BLASTp.

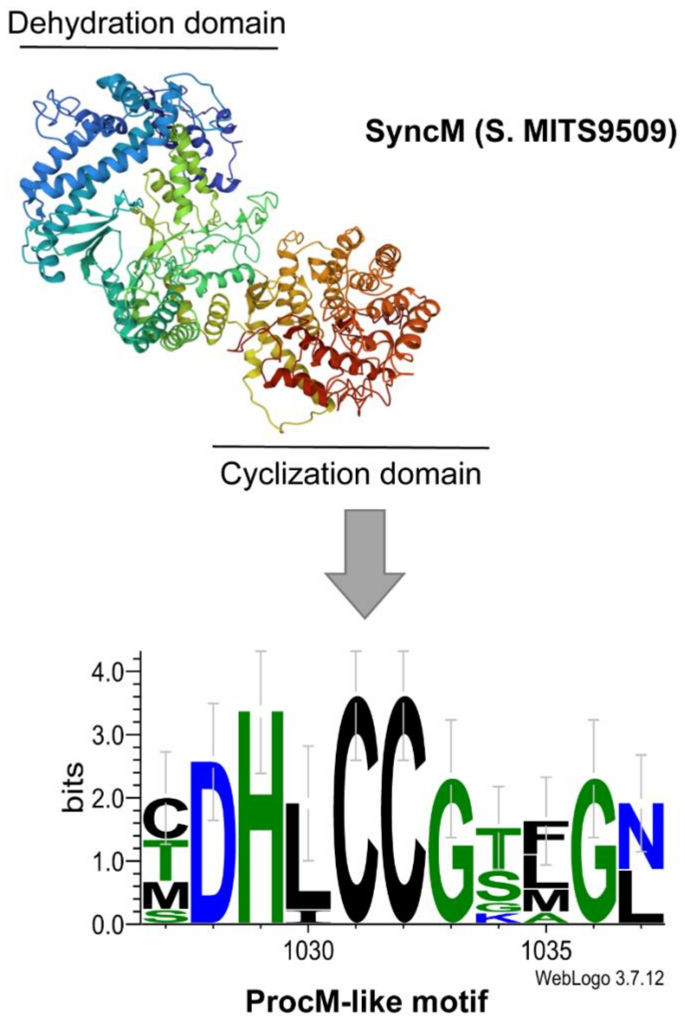

Figure S4. I-TASSER model of Sequence logo of the conserved "CCG" motif characteristic in the cyclization domain of ProcM-like enzymes adapted Arias-Orozco P. et al, (2023) (5). All nine ProcM enzymes were aligned in MEGA X using ClustalW, and a sequence logo was generated at WebLogo 3 (4).

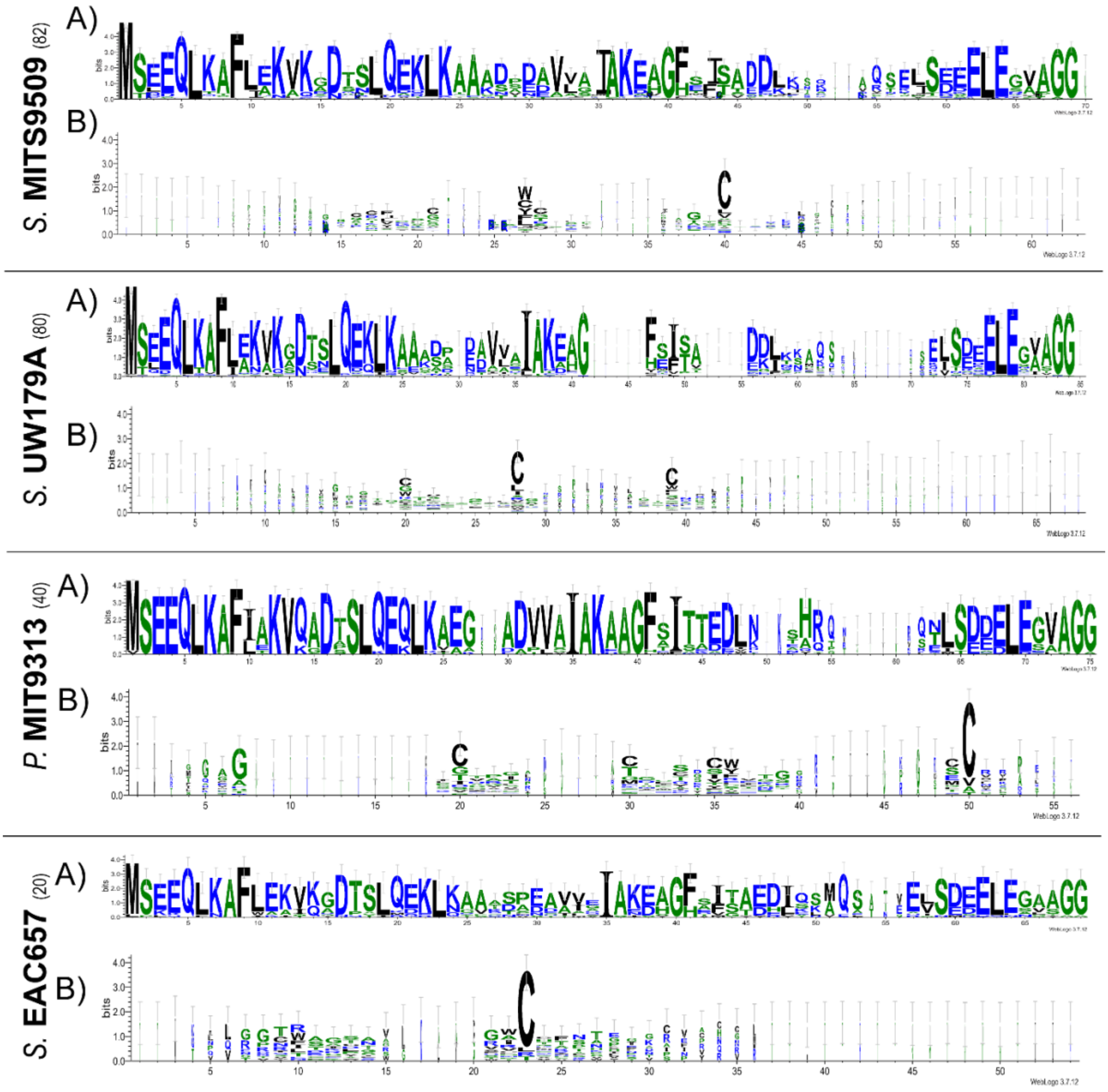

Figure S5. Prochlorosins sequence logo (4) found in each strain. A) Conserved Leader core peptide N-terminal and B) Hypervariable core peptide.

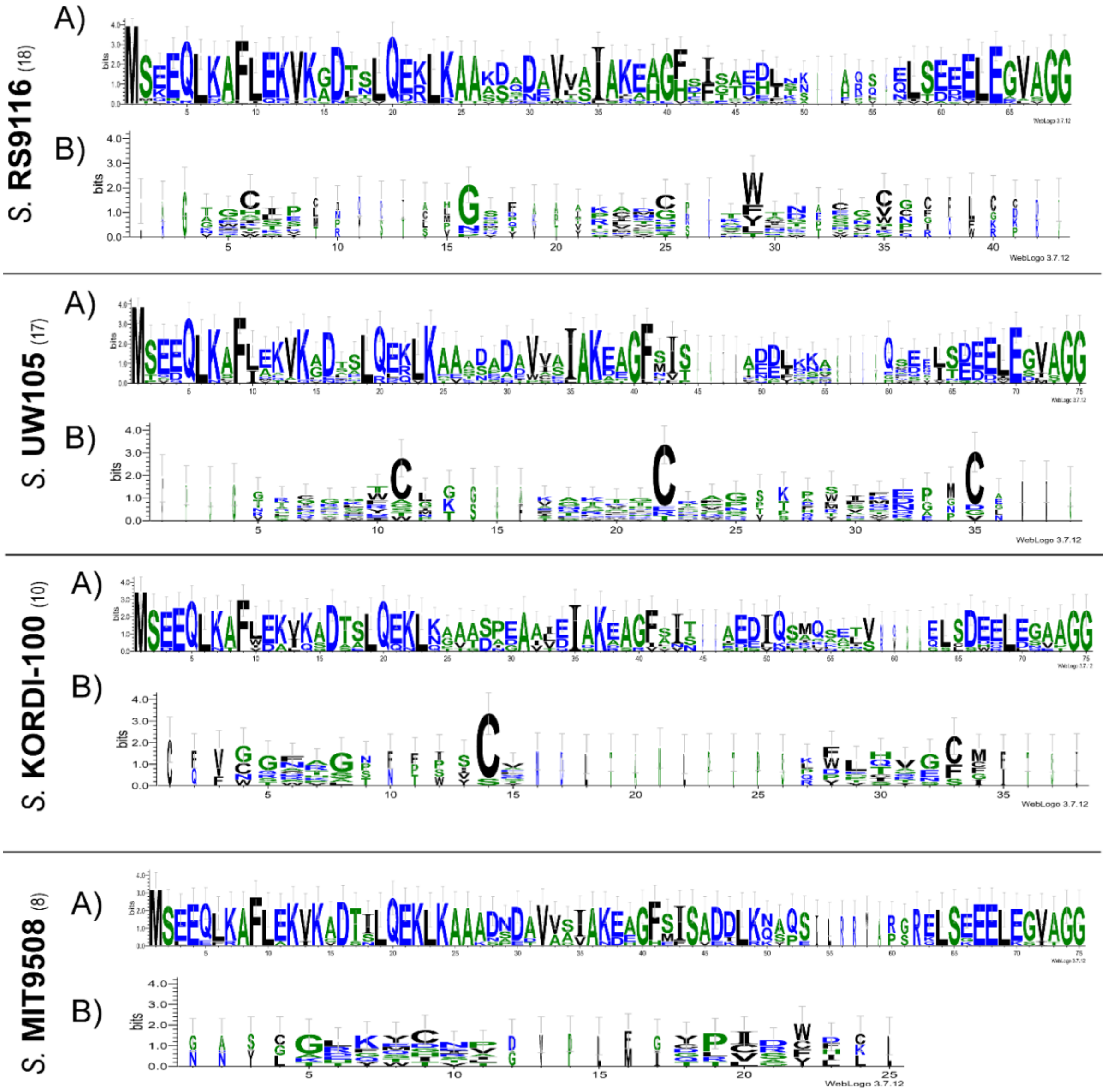

Cont. Figure S5. Prochlorosins sequence logo (4) found in each strain. A) Conserved Leader core peptide N-terminal and B) Hypervariable core peptide.

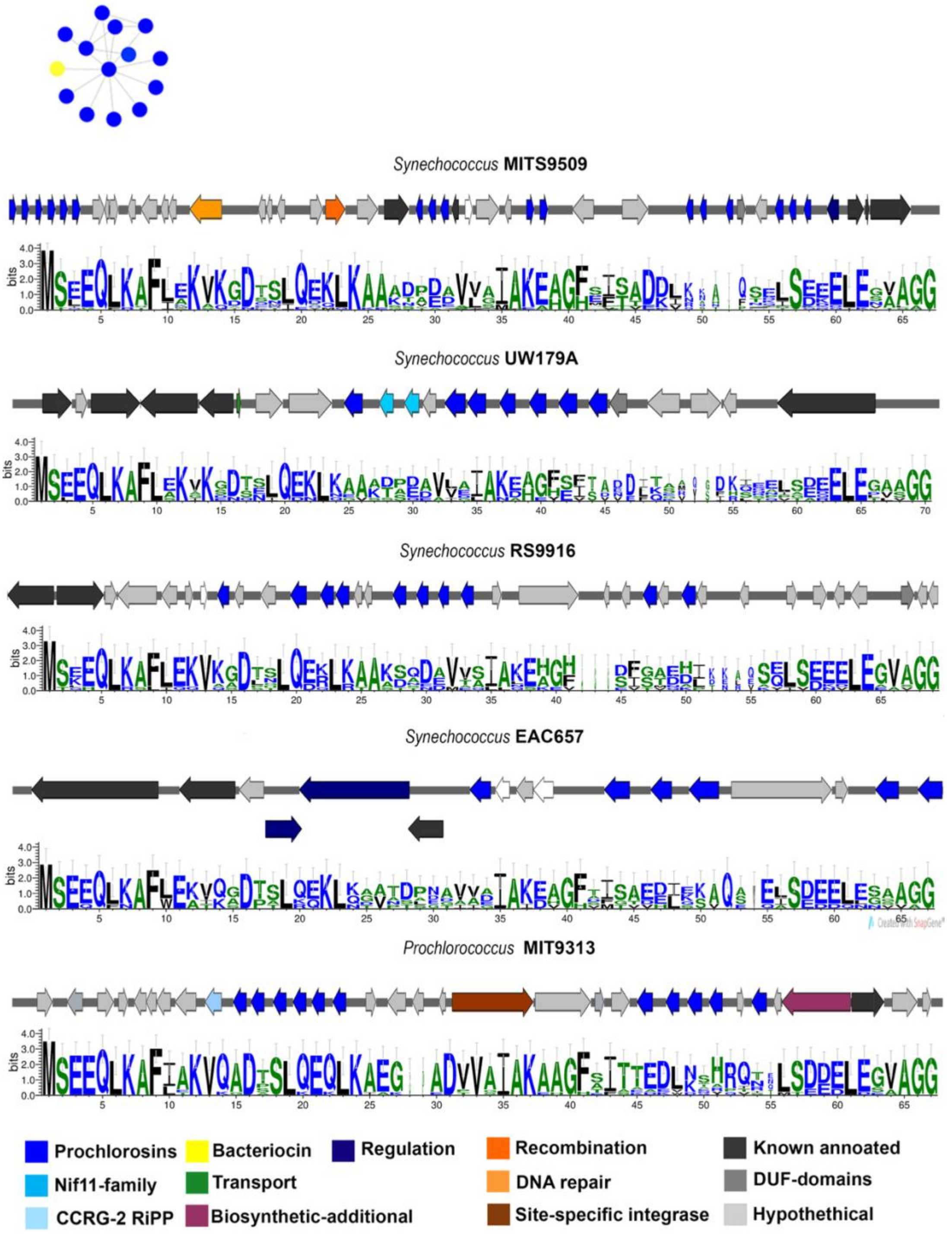

Figure S6. Representative of BGCs contained prochlorosins in tandem (GCFs group A). The sequence logo (4) for the conserved predicted leader peptide found in each biosynthetic cluster is shown.

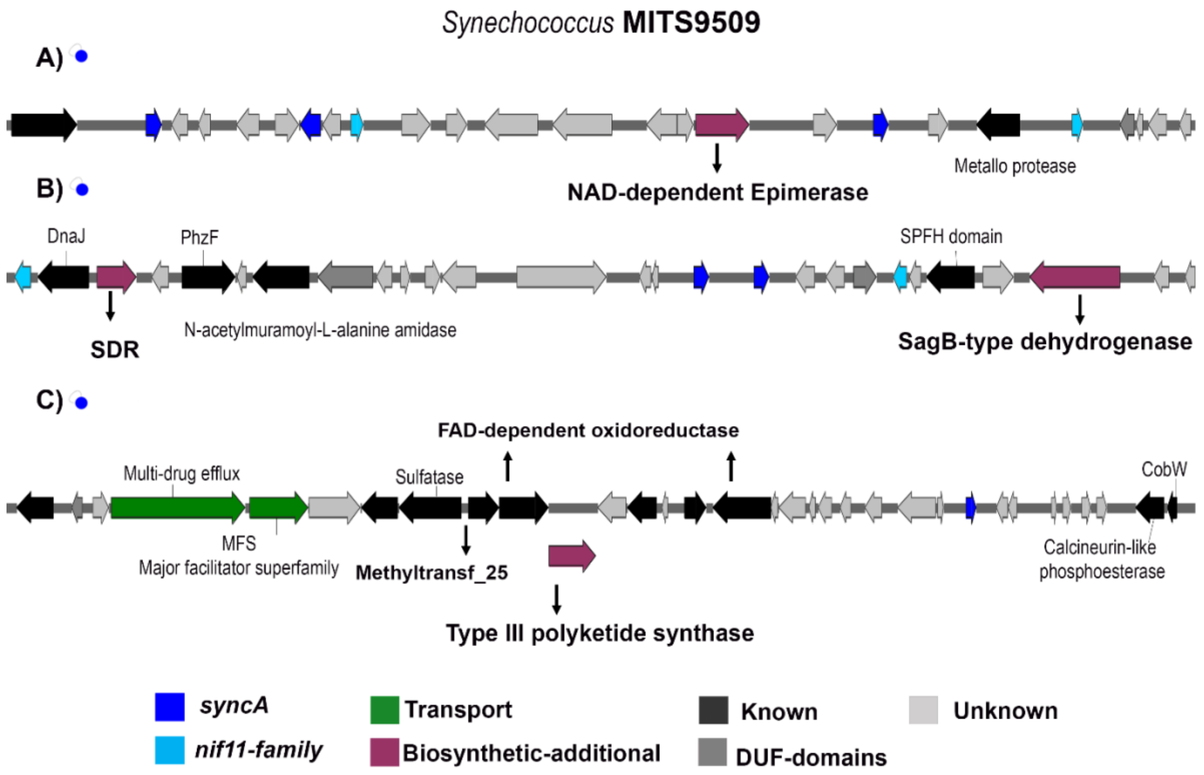

Figure S7. Continuation of *Synechococcus* MITS9509 Prochlorosin genomic cluster context. Examples of singletons BGC with additional annotated biosynthetic enzymes and other neighboring proteins with encoded prochlorosins. BGC-*sync*-containing with A) NAD-dependent epimerase B) SagB-type dehydrogenase and SDR: Short chain oxidoreductase .C) BGC Synechococcsins + Type III PKS.

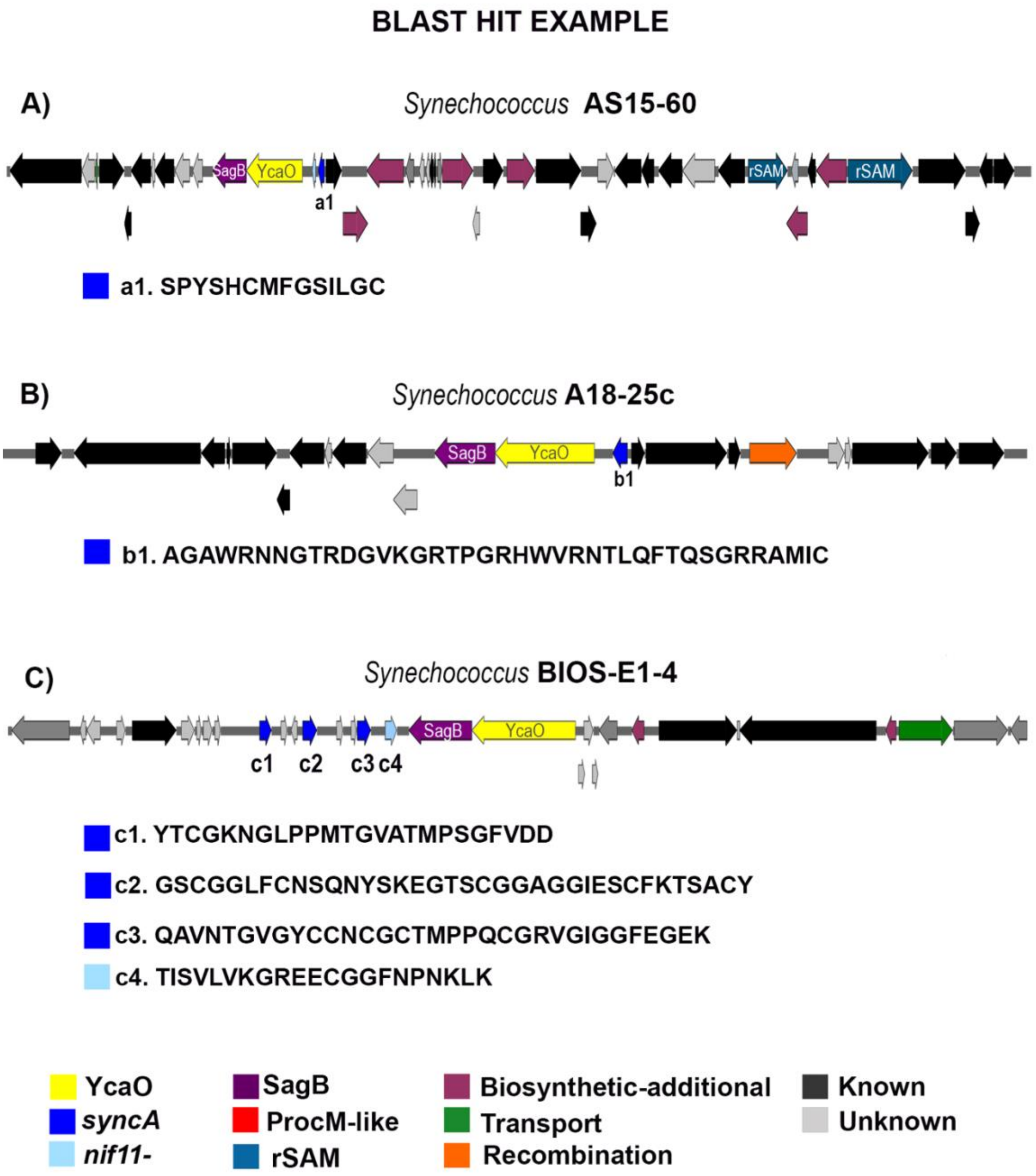

Figure S8. Novel BGCs of a putative LAP/Thiopeptide identified regions in *Synechococcus* strains harboring putative *syncA* and other *nif11* precursor family genes identified after BLAST analysis of the YcaO amino acid sequence from *S.* UW179A. Different SyncA putative peptide core sequences are indicated in lowercase.

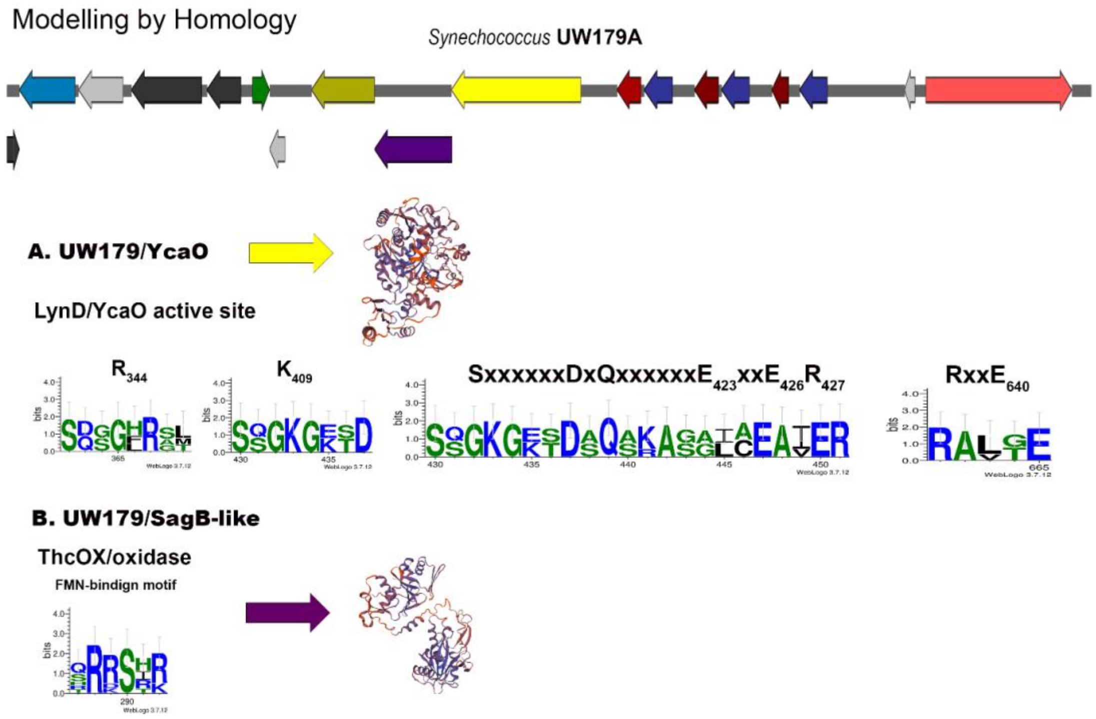

Figure S9. Analysis of the new S. UW179A YcaO cluster by homology modeling with ExpAsy SWISS-MODEL (6-12). A) YcaO model had a QMEAN of 0.63±0.05. The homology model was based on LynD and TruD from cyanobactin pathways. Sequence identity was (~26%). Sequence logo of the conserved YcaO domain ATP binding site (13, 14) . B) SagB-like model had a QMEAN of 0.66 ±0.05. With ~26% identity to the oxidase from the cyanobactin pathway. ThcOx (15) A sequence logo of A known FMN-binding motif other dehydrogenases/oxidases (16).

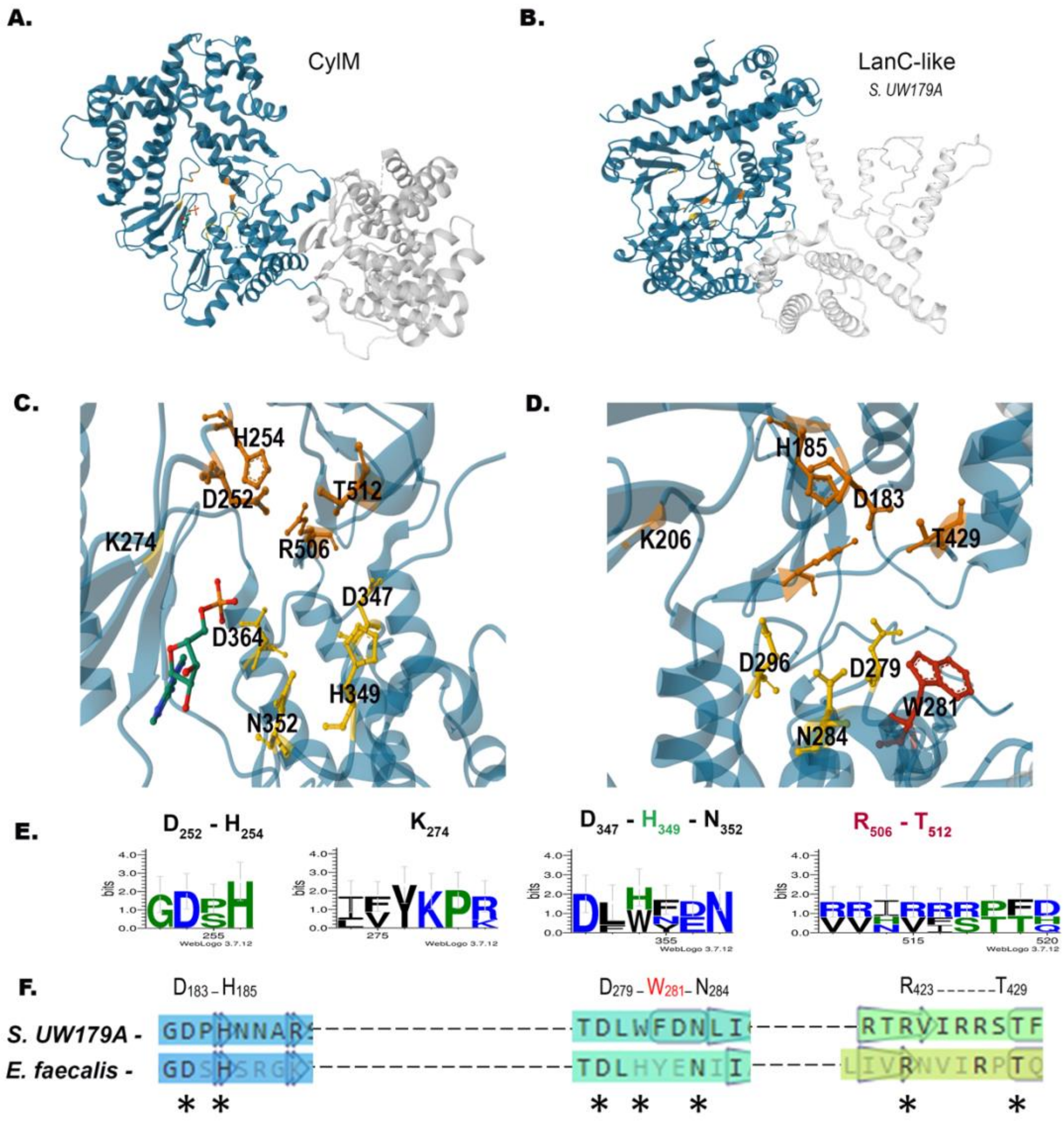

Figure S10. LanC-like model prediction analysis of *S.* UW179A and comparison with CylM (6-12). A. CylM crystal structure (18) (**PDB-5dzt.1**). Colored domains indicated: Dehydration/Blue and Cyclization/Light gray. **B.** LanC-like homology model was based on AlphaFold predicted structure putative type II lanthionine synthetase *S.* BIOS-E4-1 (A0A7G8DBZ6.1 - identity 95.43%). **C.** CylM active site. Residues in yellow are proposed to be involved in phosphorylation. Residues in orange are involved in the elimination step .**D.** The proposed active site of LanC-like (**$SynB_{like-UW179}$**). **E.** Sequence logo of the conserved active site using CylM (17,18) alignment (identity 16.83%). H_{349} is a W_{281}, indicated in green.

Red residues indicate correct homology alignment in ExpAsy. **F.** Sequence Alignment in ExpAsy indicating residues of interest in the dehydration domain (*S.* UW179A LanC-like and *E. faecalis* CylM).

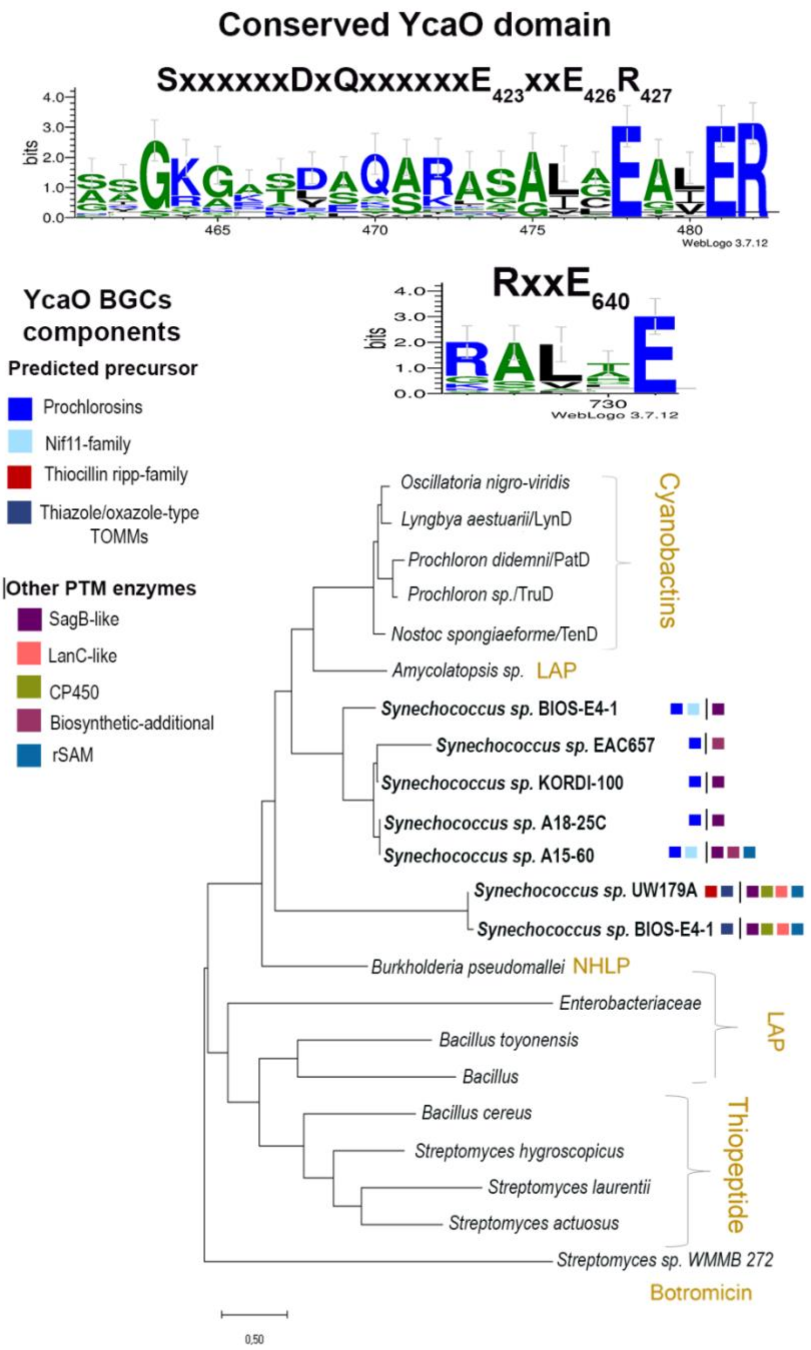

Figure S11. Neighbor-Joining Phylogenetic three of YcAO enzymes identified and reference sequence (19-21). Sequence logo of the conserved YcaO domain ATP binding site including all the identified enzymes in this study (13, 14). *Synechococcus* strains with the new YcaO biosynthetic gene cluster are underlined. The colored squares identify other components of the analyzed BGC cluster.

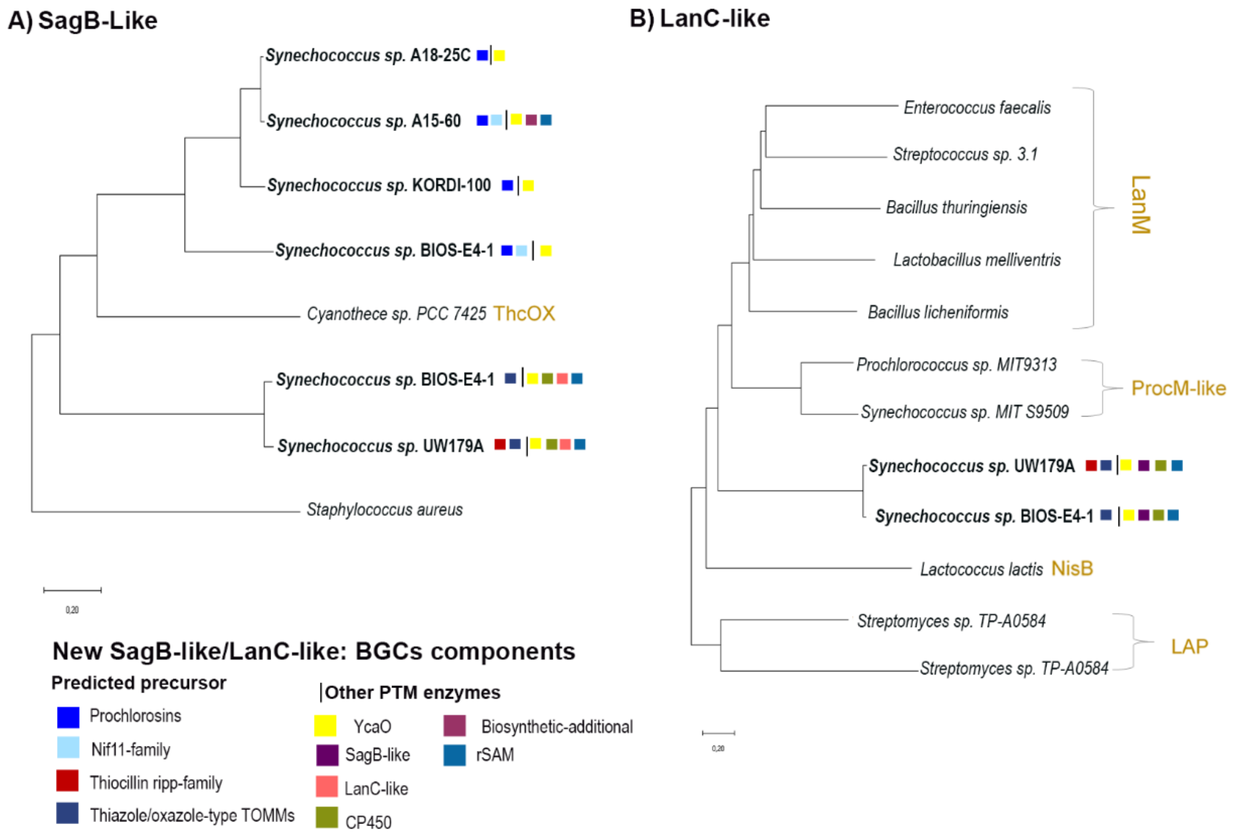

Figure S12. Neighbor-Joining Phylogenetic tree of SagB-like and Lan-C-like enzymes identified and reference sequence (19-21). *Synechococcus* strains with the new biosynthetic gene cluster are underlined. The colored squares identify other components of the analyzed BGC cluster.

REFERENCE

1. Cubillos-Ruiz A, Berta-Thompson JW, Becker JW, van der Donk WA, Chisholm SW. 2017. Evolutionary radiation of lanthipeptides in marine cyanobacteria. Proc Natl Acad Sci U S A 114:E5424-E5433.
2. Lee MD, Ahlgren NA, Kling JD, Walworth NG, Rocap G, Saito MA, Hutchins DA, Webb EA. 2019. Marine *Synechococcus* isolates representing globally abundant genomic lineages demonstrate a unique evolutionary path of genome reduction without a decrease in GC content. Environ Microbiol 21:1677-1686.
3. Dore H, Farrant GK, Guyet U, Haguait J, Humily F, Ratin M, Pitt FD, Ostrowski M, Six C, Brillet-Gueguen L, Hoebeke M, Bisch A, Le Corguille G, Corre E, Labadie K, Aury JM, Wincker P, Choi DH, Noh JH, Eveillard D, Scanlan DJ, Partensky F, Garczarek L. 2020. Evolutionary Mechanisms of Long-Term Genome Diversification Associated With Niche Partitioning in Marine Picocyanobacteria. Front Microbiol 11:567431.
4. Crooks GE, Hon G, Chandonia JM, Brenner SE. 2004. WebLogo: a sequence logo generator. Genome Res 14:1188-90.
5. Arias-Orozco P, Inklaar M, Lanooij J, Cebrian R, Kuipers OP. 2021. Functional Expression and Characterization of the Highly Promiscuous Lanthipeptide Synthetase SyncM, Enabling the Production of Lanthipeptides with a Broad Range of Ring Topologies. ACS Synth Biol 10:2579-2591.
6. Waterhouse A, Bertoni M, Bienert S, Studer G, Tauriello G, Gumienny R, Heer FT, de Beer TAP, Rempfer C, Bordoli L, Lepore R, Schwede T. 2018. SWISS-MODEL: homology modelling of protein structures and complexes. Nucleic Acids Res 46:W296-w303.

7. Studer G, Biasini M, Schwede T. 2014. Assessing the local structural quality of transmembrane protein models using statistical potentials (QMEANBrane). Bioinformatics 30:i505-i511.

8. Bienert S, Waterhouse A, de Beer Tjaart AP, Tauriello G, Studer G, Bordoli L, Schwede T. 2016. The SWISS-MODEL Repository—new features and functionality. Nucleic Acids Research 45:D313-D319.

9. Guex N, Peitsch MC, Schwede T. 2009. Automated comparative protein structure modeling with SWISS-MODEL and Swiss-PdbViewer: A historical perspective. ELECTROPHORESIS 30:S162-S173.

10. Studer G, Tauriello G, Bienert S, Biasini M, Johner N, Schwede T. 2021. ProMod3—A versatile homology modelling toolbox. PLOS Computational Biology 17:e1008667.

11. Studer G, Rempfer C, Waterhouse AM, Gumienny R, Haas J, Schwede T. 2019. QMEANDisCo—distance constraints applied on model quality estimation. Bioinformatics 36:1765-1771.

12. Benkert P, Biasini M, Schwede T. 2011. Toward the estimation of the absolute quality of individual protein structure models. Bioinformatics 27:343-50.

13. Koehnke J, Mann G, Bent AF, Ludewig H, Shirran S, Botting C, Lebl T, Houssen W, Jaspars M, Naismith JH. 2015. Structural analysis of leader peptide binding enables leader-free cyanobactin processing. Nat Chem Biol 11:558-563.

14. Dunbar KL, Chekan JR, Cox CL, Burkhart BJ, Nair SK, Mitchell DA. 2014. Discovery of a new ATP-binding motif involved in peptidic azoline biosynthesis. Nat Chem Biol 10:823-9.

15. Bent AF, Mann G, Houssen WE, Mykhaylyk V, Duman R, Thomas L, Jaspars M, Wagner A, Naismith JH. 2016. Structure of the cyanobactin oxidase ThcOx from

Cyanothece sp. PCC 7425, the first structure to be solved at Diamond Light Source beamline I23 by means of S-SAD. Acta Crystallogr D Struct Biol 72:1174-1180.

16. Melby JO, Li X, Mitchell DA. 2014. Orchestration of enzymatic processing by thiazole/oxazole-modified microcin dehydrogenases. Biochemistry 53:413-22.

17. Repka LM, Chekan JR, Nair SK, van der Donk WA. 2017. Mechanistic Understanding of Lanthipeptide Biosynthetic Enzymes. Chem Rev 117:5457-5520.

18. Dong, S. H., Tang, W., Lukk, T., Yu, Y., Nair, S. K., and van der Donk, W. A. (2015) The enterococcal cytolysin synthetase has an unanticipated lipid kinase fold, Elife 4.

19. Saitou N, Nei M. 1987. The neighbor-joining method: a new method for reconstructing phylogenetic trees. Mol Biol Evol 4:406-25.

20. Tamura K, Stecher G, Kumar S. 2021. MEGA11: Molecular Evolutionary Genetics Analysis Version 11. Mol Biol Evol 38:3022-3027.

21. Bryson V, Vogel HJ. 1965. Evolving Genes and Proteins. Science 147:68-71.
